# Metabolomic fingerprinting and systemic inflammatory profiling of asthma COPD overlap (ACO)

**DOI:** 10.1186/s12931-020-01390-4

**Published:** 2020-05-24

**Authors:** Nilanjana Ghosh, Priyanka Choudhury, Sandeep Rai Kaushik, Rakesh Arya, Ranjan Nanda, Parthasarathi Bhattacharyya, Sushmita Roychowdhury, Rintu Banerjee, Koel Chaudhury

**Affiliations:** 1 School of Medical Science and Technology, Indian Institute of Technology Kharagpur, Kharagpur, 721302 India; 2 Translational Health Group, International Centre for Genetic Engineering and Biotechnology, New Delhi, India; 3 Institute of Pulmocare and Research, Kolkata, India; 4 Apollo Gleneagles Hospital, Kolkata, India; 5 Department of Agricultural and Food Engineering, Indian Institute of Technology Kharagpur, Kharagpur, India

**Keywords:** Asthma COPD overlap (ACO), Metabolomics, Mass spectrometry, Inflammatory mediators

## Abstract

**Background:**

Asthma-COPD overlap (ACO) refers to a group of poorly studied and characterised patients reporting with disease presentations of both asthma and COPD, thereby making both diagnosis and treatment challenging for the clinicians. They exhibit a higher burden in terms of both mortality and morbidity in comparison to patients with only asthma or COPD. The pathophysiology of the disease and its existence as a unique disease entity remains unclear. The present study aims to determine whether ACO has a distinct metabolic and immunological mediator profile in comparison to asthma and COPD.

**Methods:**

Global metabolomic profiling using two different groups of patients [discovery (D) and validation (V)] were conducted. Serum samples obtained from moderate and severe asthma [*n* = 34(D); *n* = 32(V)], moderate and severe COPD [*n* = 30(D); 32(V)], ACO patients [*n* = 35(D); 40(V)] and healthy controls [*n* = 33(D)] were characterized using gas chromatography mass spectrometry (GC-MS). Multiplexed analysis of 25 immunological markers (IFN-γ (interferon gamma), TNF-α (tumor necrosis factor alpha), IL-12p70 (interleukin 12p70), IL-2, IL-4, IL-5, IL-13, IL-10, IL-1α, IL-1β, TGF-β (transforming growth factor), IL-6, IL-17E, IL-21, IL-23, eotaxin, GM-CSF (granulocyte macrophage-colony stimulating factor), IFN-α (interferon alpha), IL-18, NGAL (neutrophil gelatinase-associated lipocalin), periostin, TSLP (thymic stromal lymphopoietin), MCP-1 (monocyte chemoattractant protein- 1), YKL-40 (chitinase 3 like 1) and IL-8) was also performed in the discovery cohort.

**Results:**

Eleven metabolites [serine, threonine, ethanolamine, glucose, cholesterol, 2-palmitoylglycerol, stearic acid, lactic acid, linoleic acid, D-mannose and succinic acid] were found to be significantly altered in ACO as compared with asthma and COPD. The levels and expression trends were successfully validated in a fresh cohort of subjects. Thirteen immunological mediators including TNFα, IL-1β, IL-17E, GM-CSF, IL-18, NGAL, IL-5, IL-10, MCP-1, YKL-40, IFN-γ, IL-6 and TGF-β showed distinct expression patterns in ACO. These markers and metabolites exhibited significant correlation with each other and also with lung function parameters.

**Conclusions:**

The energy metabolites, cholesterol and fatty acids correlated significantly with the immunological mediators, suggesting existence of a possible link between the inflammatory status of these patients and impaired metabolism. The present findings could be possibly extended to better define the ACO diagnostic criteria, management and tailoring therapies exclusively for the disease.

## Background

Asthma and chronic obstructive pulmonary disease (COPD) are two heterogenous obstructive airway disorders that are associated with distinct pathological mechanisms. Asthma is broadly characterized by airway hyperresponsiveness which leads to reversible airflow obstruction based primarily on type 2 eosinophilic inflammation [1, 2]. COPD shows progressive and irreversible airflow obstruction typically caused by exposure to noxious gases and is majorly associated with neutrophilic inflammation involving CD8^+^lymphocytes and macrophages [1, 3]. Asthma-COPD overlap (ACO), frequently encountered in medical practice, refers to patients presenting with characteristics of both asthma and COPD, thereby making both diagnosis and treatment challenging for the clinicians [4].

The universally accepted definition of ACO remains elusive till date. In fact, the definition of ACO is still evolving, and different clinical definitions are being provided in various studies [5–8]. The prevalence of ACO depends on how it is defined, but it is relatively common in clinical practice, affecting 15 to 20% of patients with asthma and COPD [9]. In general, patients with ACO are reported to have poorer quality of life, rapid decline in lung function, higher frequency of exacerbations, higher mortality and morbidity in comparison to patients with only asthma or COPD [10]. ACO patients have been largely excluded from basic research and pivotal therapeutic trials, as a result of which the pathogenesis of ACO, including underlying inflammation patterns, remains poorly understood [9].

The local and systemic responses are highly activated in both asthma and COPD [11, 12]. There are reports of markers such as NGAL, YKL-40, IL-6, periostin being studied in ACO in recent years [13, 14]. Multiplexed analysis of immunological markers allows for the quantitative measurement and comparison of a broad range of inflammatory mediators that aids in creating a better understanding of the immune response under any biological condition. Heightened immune response and inflammation are reported to be associated with shift in tissue metabolism [15, 16]. The altered metabolism is a result of the recruitment of inflammatory cell types, particularly myeloid cells such as neutrophils and monocytes. This leads to the generation of large quantities of reactive nitrogen and oxygen intermediates, depletion of nutrients and increased oxygen consumption. The migration of myeloid cells to the site of inflammation is an energy consuming process and demands a large amount of ATP. Further, at the site of inflammation there is an increased nutrient, energy and oxygen demand to accomplish the process of phagocytosis [15]. This can further alter cellular metabolism, including extracellular metabolic pathways which generates biologically active molecules capable of initiating and modulating inflammatory responses [17].

Understanding the metabolic implications of chronic inflammatory processes is, therefore, an urgent need. A suitable tool for this purpose is metabolic profiling, as it allows the investigation of a broad range of small molecules (metabolites) in various body fluids. Metabolites are the intermediate and end products of cellular metabolic processes within an organism under any given physiological condition [18]. Metabolomics deals with the analysis of these metabolites present in human specimens in various states of health and disease [18]. The most commonly used samples are serum, urine, sputum, saliva and faeces, because they are obtained from patients involving minimally/non-invasive procedures [19, 20]. Advancement of analytical techniques such as nuclear magnetic resonance (NMR) and mass spectrometry (MS) has enabled quantitative identification of a wide range of metabolites using a small volume of sample.

Metabolic profiling has been successfully applied to obtain an in-depth understanding of the pathophysiology of obstructive lung diseases, such as asthma and COPD [21–24]. The precise involvement of metabolites in the pathobiology of ACO, however, is yet not well understood. Limited reports exist on metabolite studies in ACO. Eicosanoids, found to be in higher levels in ACO and metabolized through lipoxygenase, is suggested to discriminate well between ACO and COPD [25]. Another study has indicated significantly increased levels of L-histidine in urine of patients with ACO as compared with asthma or COPD [26].

Our earlier findings using NMR metabolomics indicate an enhanced energy and metabolic burden associated with ACO as compared to asthma and COPD [27]. This motivates us to gain a deeper insight into inflammation-related metabolism in ACO. The present study combines gas-chromatography-mass spectrometry (GC/MS) based metabolomics coupled with wide spectrum profiling of inflammatory mediators for this purpose.

## Materials and methods

### Subject selection

All patients were recruited at the Institute of Pulmocare and Research (IPCR) Kolkata, India. The Institutional Human Ethics Committee of IPCR, Kolkata approved this study. Written informed consent was obtained from all participants who volunteered to participate in this study. The detailed inclusion and exclusion criteria are discussed elsewhere [27]. Briefly, the recruited subjects were assigned to four groups: (a) moderate or severe cases of asthma, diagnosed based on the GINA guidelines (GINA 2014) [28] (b) stage II and III, i.e. moderate and severe COPD patients diagnosed according to the GOLD criterion (GOLD 2014) [29] (c) major criteria used for ACO diagnosis were (i) persistent airflow limitation (post-bronchodilator FEV_1_/FVC <  0.70) in individuals 40 years of age or older (ii) ≥ 10 pack-years of tobacco smoking (iii) documented history of asthma before 40 years of age, or bronchodilator response (BDR) of > 400 mL in FEV_1_; minor criteria were (i) documented history of atopy or allergic rhinitis (ii) BDR of FEV_1_ ≥ 200 mL and 12% from baseline values on two or more visits (iii) peripheral blood eosinophil count of ≥ 300 cells/μL; all major criteria and at least one minor criterion was considered for inclusion of subjects into the ACO cohort [5, 30] (d) age-matched healthy male smokers as controls having normal lung function. Only current or former male smokers were recruited in this study to avoid gender and smoking induced bias. Patients who have had history of exacerbations, active respiratory infections, had received oral corticosteroid treatment or antibiotics/antiviral drugs during the previous 3 months were excluded. Patients having other comorbidities including metabolic diseases were also excluded from this study.

The pilot metabolomic study was conducted on two different patient cohort, comprising of the discovery and validation phase. The discovery phase patient cohort consisted of (i) controls = 33 (ii) asthma = 34 (iii) COPD = 30 and (iv) ACO = 35. For the validation phase, (i) asthma = 32 (ii) COPD = 32 and (iii) ACO = 40 patients were considered. Only the discovery cohort was considered for immunological profiling. Both, the discovery and validation cohort of patients had the same inclusion and exclusion criteria.

### Sample collection

Five ml of venous blood samples were collected from subjects post confirmation of their disease status. Samples were incubated at room temperature for 45 min to allow clotting and centrifuged at 1500×g at 4 °C for 15 min. The serum fraction was separated, aliquoted, and stored immediately at − 80 °C. All samples were collected following a minimum of 12 h overnight fasting.

### GC-MS based untargeted metabolomics

#### Randomization and quality control (QC)

Coded samples were randomized using a web-based tool (www.randomizer.org) to process these samples for metabolite extraction and derivatization in batches followed by GC-MS data acquisition within 24 h. In metabolomics, use of QC samples in the quality assurance procedure provides a mechanism to assess the analytical variance of the data. The QC sample qualitatively and quantitatively represents pooled samples of equal volume obtained from all enrolled participants. These samples provide an average of all of the metabolomes analysed in the study and ensure data reproducibility [31–33].

#### Metabolite extraction and chemical derivatization for metabolomics

Each batch consisting of six test and two QC samples was thawed on ice, before metabolite extraction and derivatization procedures, as described previously [34] with minor modifications. In brief, 50 μl of serum sample was thawed on ice and 10 μl of freshly prepared isopropyl maleic acid (1 mg/ml) was added as an internal standard. Next, 800 μl of ice-cold methanol was mixed with the sample and vortexed for 30 s. The suspension was then centrifuged at 15,000×g for 10 min at 4 °C and supernatant dried in a vacuum evaporator at 40 °C for 30 min. Dried samples were then treated with 2% methoxyamine HCl in pyridine (MOX) reagent at 60 °C for 2 h followed by a silylation step with N,O-Bis (trimethylsilyl) trifluoroacetamide (BSTFA) at 60 °C for 1 h. After derivatization, the sample tubes were centrifuged at 10,000×g for 5 min and the supernatant transferred into a glass vial insert kept inside a 2 ml screw capped glass GC vial. Metabolomics standards initiative (MSI) guidelines were followed while performing all the metabolomics experiments [35]. The detailed methodology of GC-MS data acquisition, pre-processing and analysis is given in the Supplementary Materials section.

### Pathway analysis, metabolite set enrichment analysis (MSEA) and receiver operator characteristic (ROC) curves of dysregulated metabolites

Using Metaboanalyst 4.0 (www.metaboanalyst.ca), the peak areas of all the identified metabolites were subjected to pathway analysis and MSEA to identify potential key significantly altered metabolic pathways (ACO vs asthma and ACO vs COPD) [36, 37]. First, to explore the metabolic pathways that are potentially dysregulated in ACO, a global metabolic pathway analysis was carried out. The default ‘global test’ and ‘relative-betweenness centrality’ for pathway enrichment and pathway topological analyses were selected, respectively. The “current 2019” Kyoto Encyclopedia of Genes and Genomes (KEGG) version pathway library was also used.

For MSEA, quantitative enrichment analysis (QEA) was performed on normalized data for comprehensive screening of affected pathways. QEA is based on the global test algorithm to perform enrichment analysis directly from raw concentration data (peak area in this case) and does not require a list of significantly changed compounds. The QEA algorithm uses a generalized linear model to estimate a ‘Q-stat’ for each metabolite set. In addition to the Q-stat values, the QEA module also provide *p*-values, Holm adjusted p-values, and estimates of false discovery rate (FDR).

The prediction ability of all significant metabolites was assessed using receiver operating characteristic (ROC) curve and the area under the curve (AUC) calculated (MedCalc Statistical Software version, version 19.2.1, MedCalc Software bvba, Ostend, Belgium).

### Clinical correlation of significantly altered metabolites with lung function parameters

The relationship between significantly altered metabolites (common to ACO vs. asthma and ACO vs. COPD) and lung function parameters of ACO subjects was explored using Pearson’s correlation analysis (GraphPad Prism version 7.00 for Windows, GraphPad Software, San Diego, CA, USA). This was done to investigate the extent to which the dysregulated metabolites were linearly related to FEV_1_ and FEV_1_/FVC of ACO subjects.

### Multiplex analysis of immunological mediators

Serum levels of human IFN-γ (interferon gamma), TNF-α (tumor necrosis factor alpha), IL-12p70 (interleukin 12p70), IL-2, IL-4, IL-5, IL-13, IL-10, IL-1β, IL-1α, TGF-β (transforming growth factor), IL-6, IL-17E, IL-21, IL-23, eotaxin, GM-CSF (granulocyte macrophage-colony stimulating factor), IFN-α (interferon alpha), IL-18, NGAL (neutrophil gelatinase-associated lipocalin), periostin, TSLP (thymic stromal lymphopoietin), MCP-1 (monocyte chemoattractant protein- 1), YKL-40 (chitinase 3 like 1) and IL-8 were measured using Magnetic Luminex Assay-Human Premixed Multi-Analyte Kit (R&D Systems Inc., Minneapolis, MN, USA) based on the Luminex xMAP technology (Luminex Corporation, Austin, Tex). The analytes IFN-γ, IL-13, IL-1β, IL-1α, TGF-β, IL-6, IL-17E, IL-21, IL-23, eotaxin, GM-CSF, IFN-α, IL-18, NGAL, periostin, TSLP and IL-22 were analyzed using a 17-plex assay. The remaining analytes TNF-α, IL-8, YKL-40, IL-10, IL-2, IL-4, IL-5, MCP-1 and IL-12p70 were part of a 9-plex assay. IL-22 was below the detection limit of the assay and hence was excluded from further analysis.

Data was read on the Luminex MAGPIX machine (Luminex Corporation) and analyzed using XPONENT 4.2 software (Luminex Corporation). The test procedure was adopted as per the manufacturer’s instructions. As per the manufacturer’s instructions, the lower detection limit of each analyte is shown in brackets: IFN-γ (0.40 pg/mL), TNF-α (1.2 pg/ml), IL-12p70 (20.2 pg/ml), IL-2 (1.8 pg/ml), IL-4 (9.3 pg/ml), IL-5 (0.5 pg/ml), IL-13 (36.6 pg/ml), IL-10 (1.6 pg/ml), IL-1α (0.9 pg/ml), IL-1β (11.1 pg/ml), IL-6 (1.7 pg/mL), IL-17E (27.7 pg/ml), IL-21 (0.869 pg/ml), IL-23 (11.4 pg/ml), eotaxin (14.6 pg/ml), GM-CSF (4.1 pg/ml), IFN-α (0.26 pg/ml), IL-18 (1.93 pg/ml), NGAL (29.2 pg/ml), periostin (95.7 pg/ml), TSLP (0.432 pg/ml), MCP-1 (9.9 pg/ml),YKL-40 (3.30 pg/ml), TGF-β (15.4 pg/ml) and IL-8 (1.8 pg/ml). Each sample was analysed in triplicate, and the mean of the three was calculated for every analyte. The standard curve was generated by a 5-parameters logistic fit.

### Statistical analysis

All values are expressed as mean ± standard deviation (SD). One way ANOVA (Dunnett’s post hoc test) or Kruskal–Wallis test (Dunn’s post hoc test) was conducted for pairwise comparisons. Statistical analyses were performed using GraphPad Prism version 7.00 for Windows, GraphPad Software, San Diego, CA, USA. A *p*-value ≤0.05 was considered to be statistically significant. Immunological markers significantly altered in ACO as compared with asthma and COPD were identified and only those dysregulated mediators common to both ACO vs. asthma and ACO vs. COPD considered for further analysis. Pearson’s correlation analysis was performed between each of these mediators and the lung function parameters, i.e. FEV_1_ and FEV_1_/FVC of ACO subjects.

### Correlation heat-maps

Correlation heatmaps using Pearson’s correlation coefficient were generated to identify the relationship between the significantly altered metabolites and immunological markers in ACO cases using R statistical packages version 3.2.2 (R Foundation for Statistical Computing, Vienna, Austria; http://www.R-project.org/).

## Results

### Untargeted GC–MS based metabolomic profile

#### Discovery phase

The baseline clinical characteristics of all subjects recruited is tabulated in Table 1. Following spectral annotation with NIST 14 library, a total of 145 consistent metabolites could be identified. Out of these metabolites, 85 had an occurrence frequency of at least 80% among all samples and were considered for further analysis. A representative GC–MS spectrum is shown in Supplementary Fig. 1. Both multivariate analysis (MVA) and univariate analysis (UVA) were performed on the constant sum normalized, log transformed and mean scaled peak area data matrix of the final 85 annotated metabolites.

**Table 1:** Clinical characteristics of the recruited subjects

	DISCOVERY COHORT					VALIDATION COHORT			
	CONTROLS	ASTHMA	COPD	ACOS	p-value	ASTHMA	COPD	ACOS	p-value
**Total Number of subjects (n)**	33	34	30	35		32	32	40	
**Age (years)**	50.90 ± 7.20	51.91 ± 7.16	54.97 ± 6.11	53.97 ± 5.89	ns	53.73 ± 5.38	55.26 ± 4.2	54.58 ± 4.6	ns
**Body Mass Index (kg/m**^**2**^**)**	21.35 ± 1.64	21.43 ± 1.62	20.78 ± 1.58	20.96 ± 1.65	ns	21.79 ± 1.59	19.13 ± 1.73	20.29 ± 1.22	ns
**Pre BD FEV1% predicted**	97.6 ± 17.8	61.3 ± 15.3	55.3 ± 14.6	52.7 ± 13.6	< 0.0001	64.4 ± 10.6	53.1 ± 12.6	50.5 ± 11.2	< 0.0001
**Post BD Lung Function**									
**FEV**_**1**_**(L)**	3.8 ± 0.2	2.2 ± 0.2	1.5 ± 0.5	1.9 ± 0.2	< 0.0001	2.4 ± 0.1	1.3 ± 0.4	2.05 ± 0.2	< 0.0001
**FVC (L)**	4.6 ± 0.3	3.2 ± 0.8	2.9 ± 0.4	3.0 ± 0.8)	< 0.0001	3.5 ± 0.2	2.7 ± 0.4	3.2 ± 0.5	< 0.0001
**FEV**_**1**_**% predicted**	99.2 ± 16.8	70.4 ± 11.3	57.6 ± 10.2	59.1 ± 9.8	< 0.0001	72.7 ± 9.7	56.9 ± 8.5	57.5 ± 6.3	< 0.0001
**FEV**_**1**_**/FVC (%)**	83.2 ± 2.1	68.8 ± 10.1	58.5 ± 6.4	56.7 ± 2.4	< 0.0001	69.1 ± 7.2	50.5 ± 7.3	61.3 ± 7.2	< 0.0001
**Smoking status (n)**									
**Former smokers**	15	21	20	13		15	21	12	
**Current smokers**	18	13	10	22		17	11	28	
**Smoking history (pack-years)**	15.3 ± 5.89	16.8 ± 4.88	35.04 ± 5.6	29.9 ± 5.7	< 0.0001	14.7 ± 3.78	37.2 ± 4.9	31.9 ± 4.7	< 0.0001
**Blood eosinophil cell/μL, median (IQR)**	120 (0–160)	340 (0–3840)	140 (0–300)	330 (0–1500)	< 0.0001	365 (0–3840)	150 (0–300)	345 (0–1500)	< 0.0001
**Frequency of exacerbation/year**	–	0.95 ± 0.20	1.78 ± 0.36	1.11 ± 0.75	< 0.0001	0.82 ± 0.11	1.87 ± 0.22	1.21 ± 0.56	< 0.0001
**Respiratory symptoms**									
**Wheezing**	–	17.4 ± 0.6	13.3 ± 0.7	25.3 ± 0.4	< 0.0001	19.1 ± 0.3	14.7 ± 0.4	27.6 ± 0.6	< 0.0001
**Expectoration**	–	12.1 ± 0.3	19.4 ± 0.3	15.6 ± 0.5	< 0.0001	10.4 ± 0.5	20.3 ± 0.6	13.8 ± 0.3	< 0.0001
**mMRC scale (0–4)**	–	–	2.4 ± 0.21	1.2 ± 0.6	< 0.0001	–	2.25 ± 0.51	1.35 ± 0.5	< 0.0001
**Atopic status/allergy n(%)**	–	29 (86)	3 (11)	24 (68)	< 0.0001	27 (83)	2 (7)	29 (72)	< 0.0001
**Treatment n(%)**									
**ICS**	–	33 (97)	17 (58)	27 (78)	< 0.0001	28 (89)	19 (60)	33 (82)	< 0.0001
**LABA**	–	25 (74)	26 (87)	29 (82)	< 0.0001	22 (70)	26 (82)	31 (78)	< 0.0001
**LAMA**	–	15.3 (45)	25 (83)	27 (76)	< 0.0001	10 (32)	24 (75)	28 (70)	< 0.0001
**PD4I**	–	–	4 (14)	3 (8)	< 0.0001	–	4 (12)	2 (5)	< 0.0001
**LTRA/theophylline**	–	24 (72)	1 (3)	16 (45)	< 0.0001	20 (63)	2 (5)	16 (39)	< 0.0001

Data are presented as mean ± SD or percentages, unless otherwise stated. *BD* bronchodilator, *FEV*_*1*_ forced expiratory volume in 1 s, *FVC* forced vital capacity, *ICS* inhaled corticosteroids, *LABA* long-acting beta agonists, *LAMA* long-acting antimuscarinics *PD4I* phosphodiesterase-4-inhibitior, *LTRA* leukotriene receptor antagonist. Differences between groups were assessed by using the 1-way ANOVA test with post hoc Tukey HSD. *p* ≤ 0.05 was considered statistically significant

**Fig. 1:**
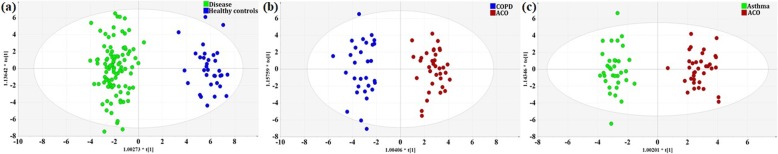
Orthogonal projections to latent structures discriminant analysis (OPLS-DA) model removes outliers which do not contribute to class separation. It is an extension to the supervised partial least squares (PLS) regression method that filters out some variance in the X-matrix unrelated to Y thereby producing results which are easier to interpret. OPLS-DA model shows optimized discrimination between (**a**) obstructive lung diseases and healthy controls (R2Y = 0.937 and Q2 = 0.919, CV-ANOVA score *p* = 0) (**b**) ACO and COPD (R2Y = 0.931 and Q2 = 0.877, CV-ANOVA score p = 0) (**c**) ACO and Asthma (R2Y = 0.95 and Q2 = 0.89, CV-ANOVA score p = 0). Parameters including R2 (goodness of the fit), Q2 (predictive ability), and analysis of variance testing of cross validated predictive residuals (CV-ANOVA) score were used to validate the robustness of the OPLS-DA model

The discovery phase samples were assigned into two groups, obstructive lung diseases (asthma, COPD and ACO) and healthy controls. MVA is a statistical technique which involves the simultaneous observation and analysis of more than two variables. Multivariate methods may be supervised or unsupervised. While unsupervised methods such as clustering are exploratory in nature and help in identification of patterns, supervised methods use some type of response variable to discover patterns associated with the response. Both PLS-DA and OPLS-DA are supervised MVA tools. PLS-DA is a chemometrics technique used to optimise separation between different groups of samples, which is accomplished by linking two data matrices X (i.e., metabolite peak areas) and Y (i.e., groups). PLS aim to differentiate between classes in highly complex data sets, despite within class variability. Initially, PLS-DA models were generated which showed class separation between the disease groups and controls (Supplementary Fig. 2). OPLS-DA is often used in lieu of PLS-DA to disentangle group-predictive and group-unrelated variation in the measured data. In doing so, OPLS-DA constructs more parsimonious and easily interpretable models compared to PLS-DA. Next, OPLS-DA models were generated for optimized separation between the two groups (Fig. 1a). The permutation test evidenced significantly higher R2 and Q2 values than that of 200 permutated models, indicating a good predictive ability of the model (Supplementary Fig. 3a).

Thereafter, PLS-DA models were generated for COPD, asthma, and ACO which exhibited good discrimination between all the three groups. This is represented as a 3D score scatter plot in Supplementary Fig. 4. Finally, for feature extraction, OPLS-DA models were generated to optimize separation for (i) ACO vs. COPD (R2X = 0.21, R2Y = 0.931 and Q2 = 0.877; CV-ANOVA score *p* = 0) (Fig. 1b) and (ii) ACO vs. asthma (R2X = 0.165, R2Y = 0.95 and Q2 = 0.89; CV-ANOVA score p = 0) (Fig. 1c). Cross-validation analysis using 200 random permutations confirms good predictive ability of both the generated models (Supplementary Fig. 3b & c). Variable of importance projection (VIP) scores were used for the identification of key metabolites contributing towards discrimination of the OPLS-DA models (Supplementary Fig. 5a & b). VIP values describe a quantitative estimation of the discriminatory power of each individual feature. Thus, VIP scores ranks the compounds (metabolites) according to their contribution to the model. Variables with VIP score > 1.3 could identify 18 metabolites for ACO vs. asthma and 17 metabolites for ACO vs. COPD responsible for clustering. Out of these, 11 metabolites [serine, threonine, ethanolamine, glucose, cholesterol, 2-palmitoylglycerol, stearic acid, lactic acid, linoleic acid, D-mannose and succinic acid] common to both ACO vs. asthma and ACO vs. COPD were considered for further analysis.

To cross-validate MVA, peak area matrix of all the metabolites were subjected to UVA using ANOVA (Dunnett’s post hoc test) or Kruskal–Wallis test (Dunn’s post hoc test), as applicable. UVA refers to statistical analyses that involve only one dependent variable and which are used to test hypotheses. UVA with multiple testing correction is an attractive approach since it is relatively simple to implement and provides a measure of statistical significance for each covariate that is easy to interpret. Common variables with VIP score > 1.3, ANOVA *p*-value ≤0.05 and adjusted FDR <  0.01 were selected as the major metabolites responsible for differentiating ACO from both, asthma and COPD. Metabolites including serine, threonine, ethanolamine, glucose, D-mannose and succinic acid were found to be down-regulated in ACO as compared to both, asthma and COPD. Cholesterol, 2-palmitoylglycerol and lactic acid were also down-regulated in ACO, but only with respect to COPD. Interestingly, these metabolites were found to be upregulated when compared with asthma. Also, two metabolites, i.e. stearic acid and linoleic acid were found to be upregulated in ACO as compared to COPD and downregulated with respect to asthma (Table 2).

**Table 2:** Human Metabolome Database identifiers (HMDB ID), multivariate data analysis (variable influence on projection (VIP) scores, false discovery rate (FDR) adjusted p-value), fold changes and pairwise univariate (ANOVA/Kruskal Wallis test) values are provided for the 11 significantly altered metabolites common to ACO vs. asthma and ACO vs. COPD. Pearson’s correlation coefficient (r) and their significance values (*p* value) depicts the association between these altered metabolites and lung function parameters, FEV1, FEV1/FVC of ACO subjects

Metabolites	HMDB ID	VIP scores			Fold change		Significance		Correlation (r)			
(Discovery cohort)		ACO vs COPD	ACO vs Asthma	Adjusted p-value (FDR)	ACO vs COPD	ACO vs Asthma	Pairwise ***p***-value		FEV1		FEV1/FVC	
							ACO vs COPD	ACO vs Asthma	Pearson (r)	p-value	Pearson (r)	p-value
Serine	HMDB0000187	1.55	2.79	< 0.0001	0.77	0.75	*	**	0.63	< 0.0001	0.54	0.0008
Threonine	HMDB0000167	2.04	2.73	< 0.0001	0.87	0.83	*	***	0.48	0.0034	0.54	0.0007
Ethanolamine	HMDB0000149	1.32	1.55	< 0.0001	0.88	0.83	*	***	0.21	ns	0.30	ns
Glucose	HMDB0000122	2.58	2.22	< 0.0001	0.76	0.73	**	***	0.67	< 0.0001	0.72	< 0.0001
Cholesterol	HMDB0000067	1.48	1.45	< 0.0001	0.85	1.29	*	**	0.59	0.0002	0.46	0.0059
2-palmitoylglycerol	HMDB0011533	2.46	1.45	< 0.0001	0.75	1.39	***	**	0.17	ns	0.52	0.0014
Stearic acid	HMDB00827	1.49	1.41	< 0.0001	1.18	0.88	*	*	−0.26	ns	−0.33	ns
Lactic acid	HMDB0000190	1.82	1.42	< 0.0001	0.83	1.18	**	*	−0.35	0.0414	−0.19	ns
Linoleic acid	HMDB0030950	1.52	1.46	< 0.0001	1.35	0.81	**	**	− 0.19	ns	− 0.52	0.0014
D-Mannose	HMDB00169	1.64	1.54	< 0.0001	0.69	0.64	**	***	0.49	0.0025	0.79	< 0.0001
Succinic acid	HMDB00254	1.42	1.82	< 0.0001	0.82	0.74	*	***	0.59	0.0002	0.69	< 0.0001

COPD- chronic obstructive pulmonary disorder, ACO- asthma COPD overlap, ns- not significant; *p ≤ 0.05; **p ≤ 0.01; ***p ≤ 0.0001

#### Validation phase

The significantly altered 11 metabolites, including serine, threonine, ethanolamine, glucose, cholesterol, 2-palmitoylglycerol, stearic acid, lactic acid, linoleic acid, D-mannose and succinic acid identified in the discovery phase were further validated in an independent fresh cohort of subjects using UVA (Table 3). Similar trends in expression with significant changes were seen as observed earlier in the discovery cohort.

**Table 3:** A new subject cohort (validation cohort) was recruited to confirm the findings of the exploratory (discovery) patient cohort. Human Metabolome Database identifiers (HMDB ID), fold changes, and pairwise univariate (ANOVA/Kruskal Wallis test) values of the 11 significantly altered serum metabolites (common to ACO vs. asthma and ACO vs. COPD) identified in the discovery cohort are summarized for the validation patient cohort

Metabolites	HMDB ID		Fold change		Significance	
(Validation cohort)		Adjusted p-value (FDR)	ACO vs COPD	ACO vs Asthma	Pairwise p-value	
					ACO vs COPD	ACO vs Asthma
L-Serine	HMDB0000187	< 0.0001	0.78	0.74	**	***
L-Threonine	HMDB0000167	< 0.0001	0.85	0.82	*	**
Ethanolamine	HMDB0000149	< 0.0001	0.86	0.79	**	***
Glucose	HMDB0000122	< 0.0001	0.81	0.76	*	**
Cholesterol	HMDB0000067	< 0.0001	0.94	1.07	*	*
2-palmitoylglycerol	HMDB0011533	< 0.0001	0.89	1.09	**	*
Stearic acid	HMDB00827	< 0.0001	1.31	0.80	**	***
Lactic acid	HMDB0000190	< 0.0001	0.81	1.29	***	**
Linoleic acid	HMDB0030950	< 0.0001	1.28	0.77	**	***
D-Mannose	HMDB00169	< 0.0001	0.79	0.79	***	***
Succinic acid	HMDB00254	< 0.0001	0.78	0.75	**	***

COPD- chronic obstructive pulmonary disorder, ACO- asthma COPD overlap, ns- not significant; *p ≤ 0.05; **p ≤ 0.01; ***p ≤ 0.0001

### Pathway analysis and MSEA

All the 85 identified and quantified metabolites in the discovery cohort were considered for pathway analysis using MetPA. All matched pathways are displayed as circles. The colour and size of each circle is based on the *p*-value and pathway impact value, respectively. Various common significantly altered pathways were observed in ACO vs. asthma and ACO vs. COPD, including starch and sucrose metabolism, linoleic acid metabolism, glycolysis / gluconeogenesis, citrate cycle (TCA cycle), glycine, serine and threonine metabolism and aminoacyl-tRNA biosynthesis (Supplementary Fig. 6a, b). A few less significant pathways could also be identified (Supplementary Table 1 and 2).

MSEA also showed multiple metabolic pathways that were significantly dysregulated in serum of ACO patients (*p* <  0.05). Supplementary Fig. 7 highlights the fold enrichment obtained when using peak areas of all the identified metabolites. The colour intensity denotes the level of statistical significance and the length of each bar represents the fold enrichment of the pathway. Supplementary Tables 3 and 4 present all the perturbed biochemical pathways with number of metabolite hits, *p*-values, Holm-adjusted p-values and FDR. Pathways with the highest number of hits and significant Holm p-values are considered to be significantly perturbed in ACO patients. The p-values of the pathways is determined by the difference of peak area data and the number of participating metabolites. Some of the most significantly altered pathways in ACO included fructose and mannose metabolism, glycine-serine-threonine metabolism, valine-leucine-isoleucine biosynthesis and glycolysis/gluconeogenesis.

### ROC curve and clinical correlation analysis of candidate metabolites in ACO

**Fig. 2:**
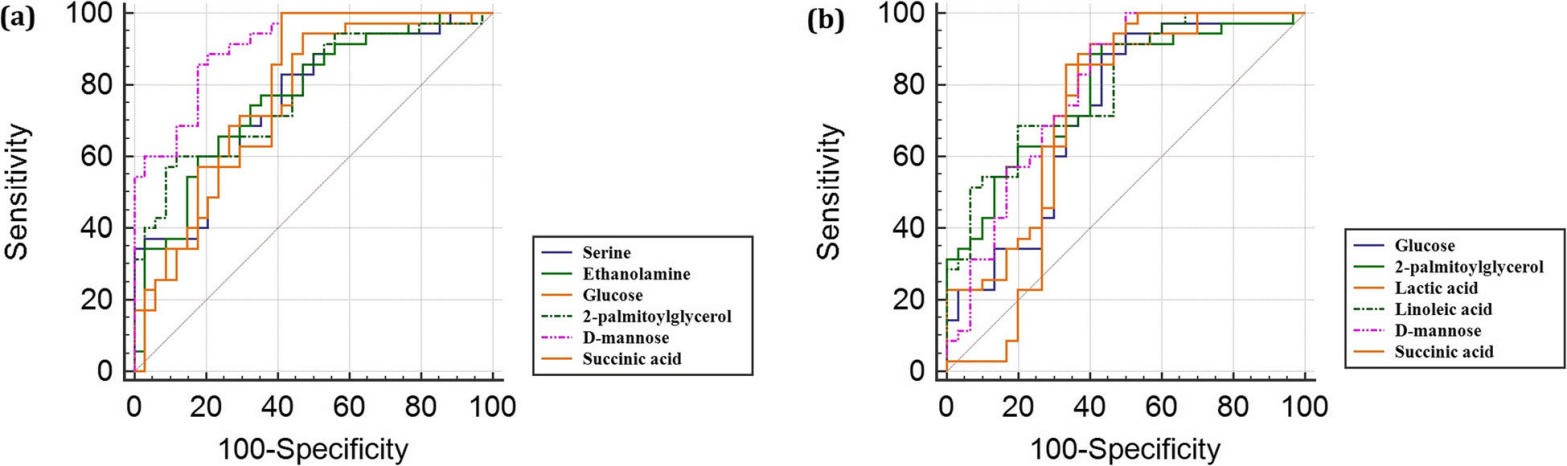
Comparison of different metabolite panels based on receiver operating characteristic (ROC) curves. The curves correspond to metabolites having the highest area under the curve (AUC) and the highest accuracy values in predicting ACO from asthma and COPD. **a** ACO versus asthma: serine, AUC = 0.753 ± 0.06 (95% confidence interval (CI) 0.634–0.849); ethanolamine, AUC = 0.767 ± 0.06 (95% CI 0.650–0.861); glucose, AUC = 0.767 ± 0.06 (95% CI 0.650–0.861); 2-palmitoylglycerol, AUC = 0.780 ± 0.06 (95% CI 0.664–0.871); D-mannose, AUC = 0.913 ± 0.03 (95% CI 0.820–0.967); succinic acid, AUC = 0.770 ± 0.06 (95% CI 0.653–0.863), **b** ACO versus COPD: glucose, AUC = 0.722 ± 0.07 (95% CI 0.597–0.826); 2-palmitoylglycerol, AUC = 0.784 ± 0.06 (95% CI 0.664–0.876); lactic acid, AUC = 0.750 ± 0.06 (95% CI 0.627–0.849); linoleic acid, AUC = 0.798 ± 0.05 (95% CI 0.680–0.888); D-mannose, AUC = 0.791 ± 0.06 (95% CI 0.673–0.882); succinic acid, AUC = 0.710 ± 0.07 (95% CI 0.584–0.816)

ROC diagram plots the true positive rate (sensitivity) of a test on the y-axis against the false positive rate (100-specificity) on the x-axis, thus producing the AUC. An AUC is a measure of the accuracy of a diagnostic test, where 1.0 indicates a perfect test and a 0.5 shows that the test is no better than random chance, and therefore has no diagnostic or prognostic value. ROC curves were generated for the common set of significantly altered metabolites of the two groups (ACO vs. asthma and ACO vs. COPD). Six metabolites with the highest AUCs (AUC > 0.7) were taken into consideration for both the groups to establish a predictive model that could well differentiate ACO from asthma and also from COPD (Fig. 2). Four metabolites viz. glucose, 2-palmitoylglycerol, D-mannose and succinic acid were found to be common between the two ROC models.

Pearson’s correlation analysis between each of the significantly altered metabolites and lung function parameters of ACO subjects is provided in Table 2. Significant positive correlation was observed between metabolites including serine, threonine, glucose, cholesterol, D-mannose, succinic acid and FEV_1_ and FEV_1_/FVC. A negative correlation was observed between stearic acid, lactic acid and linoleic acid; however, all correlations were not statistically significant.

### Serum immunological mediators and clinical correlation

Twenty-five immunological mediators (TNF-α, IFN-α, IL-1β, IL-17E/IL-25, GM-CSF, IL-18, NGAL, IL-5, IL-10, MCP-1, YKL-40, IFN-γ, IL-6, TGF-β, IL-12p70, IL-2, IL-4, IL-13, IL-1α, IL-21, IL-23, periostin, TSLP, IL-8 and eotaxin) estimated in serum of ACO, asthma, COPD and controls are shown in Supplementary Table 5. The mediators which showed significant differences in ACO with respect to asthma, COPD and controls were only considered for further analysis.

**Fig. 3:**
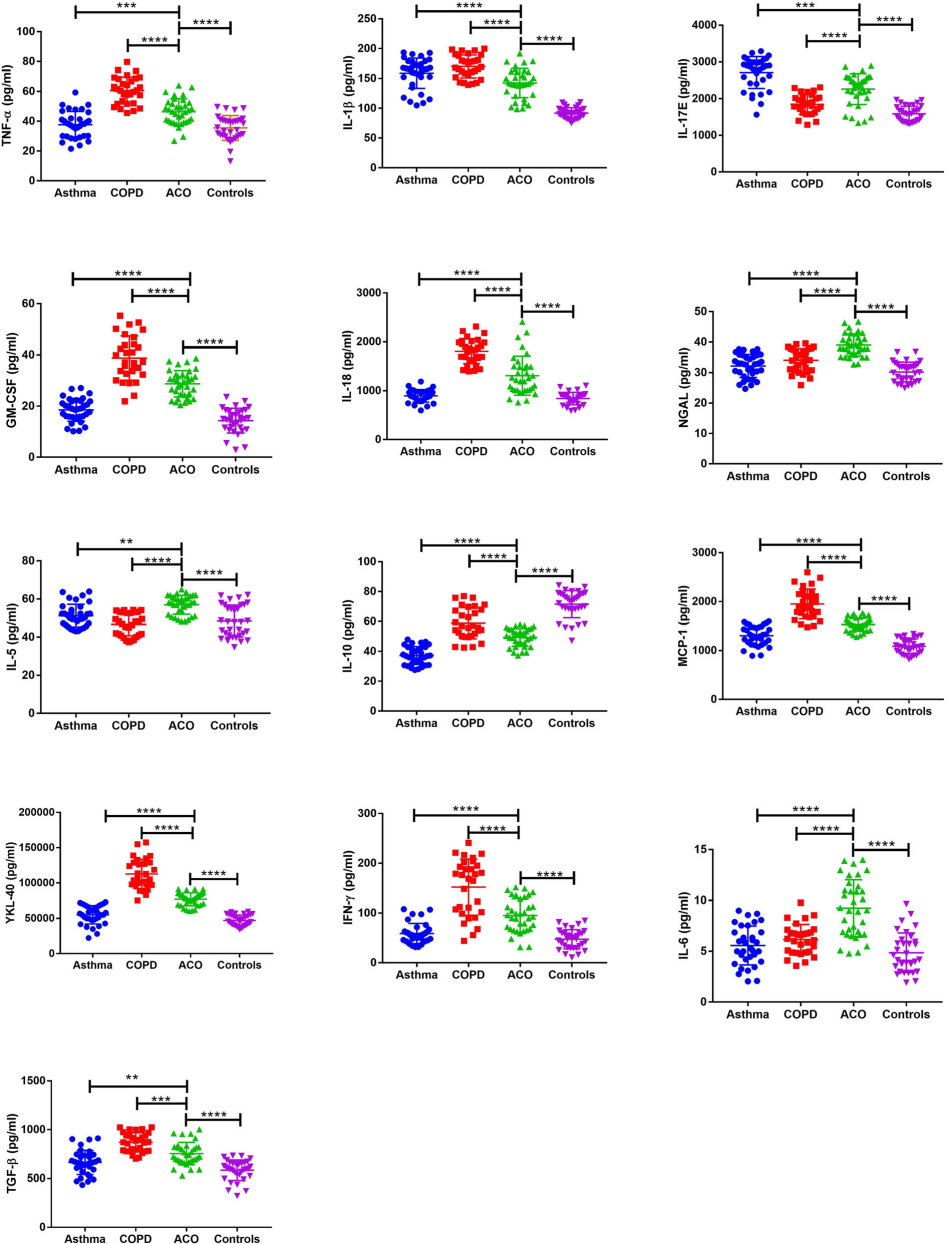
Dot plots of immunological mediator concentrations for asthma, COPD, ACO and healthy controls (TNF-α, IL-1β, IL-17E, GM-CSF, IL-18, NGAL, IL-5, IL-10, MCP-1, YKL-40, IFN-γ, IL-6 and TGF-β) which were significantly dysregulated in ACO with respect to asthma, COPD and controls. Each graph represents the concentration of a particular immunological mediator in the serum of the patients belonging to the four groups of the discovery cohort. The scatter dot plot shows the mean and standard deviation. **Notes:** ns – not significant, * *p* ≤ 0.05, **p ≤ 0.01, **p ≤ 0.001, ****p ≤ 0.0001. **Abbreviations:** ACO-Asthma COPD overlap, COPD- Chronic obstructive pulmonary disease, TNF α- Tumor necrosis factor α, IL-1β-Interleukin 1β, GM-CSF-Granulocyte-macrophage colony-stimulating factor, NGAL-Neutrophil gelatinase-associated lipocalin, MCP 1-Monocyte Chemoattractant Protein-1, YKL 40-Chitinase-3-like protein 1, IFN γ-Interferon γ, TGF β- Transforming growth factor β

The expression level of Th1 mediated cytokines, such as TNFα, and IL-1β were significantly higher in ACO cases with respect to asthma and controls. The highest expression of these cytokines was noted in patients with COPD. The expression of IL-5, a pro-inflammatory Th2 cytokine, was highest in patients with ACO as compared to asthma, COPD and controls. The anti-inflammatory cytokine, IL-10 was least expressed in asthma; the levels in ACO cases were significantly less when compared with COPD and controls. Key immunological markers, such as IFN-γ, IL-6, TGF-β and IL-17E/IL-25 showed significantly altered expression profiles in ACO. Immune system-related proteins and chemokines such as NGAL, YKL-40, MCP-1, GM-CSF etc. some of which have already been explored in ACO subjects also exhibited differential expression patterns (Fig. 3). The expression level of IFN-α could not be determined. Except IL-10, Pearson’s correlation analysis showed negative correlation between the dysregulated mediators and lung function parameters. However, the correlation was observed to be significant only for GM-CSF, IL-6, IFN-γ, YKL-40, IL-1β, NGAL and IL-5 (Table 4).

**Table 4:** Pearson’s correlation value (r) and significance values (p) depicting the extent to which the 13 dysregulated immunological mediators (significantly altered in ACO vs. asthma, ACO vs. COPD and ACO vs controls) are linearly related to FEV1 and FEV1/FVC of ACO subjects

Marker	Correlation			
	ACO vs FEV1		ACO vs FEV1/FVC	
	Pearson (r)	p-value	Pearson (r)	p-value
TNFα	−0.3717	0.0279	−0.2744	ns
IL-1β	−0.4362	0.0088	−0.6578	< 0.0001
IL-17E	−0.04455	ns	−0.3067	ns
GM-CSF	−0.09277	ns	−0.3762	0.0259
IL-18	−0.3219	ns	−0.1835	ns
NGAL	−0.3559	0.0359	−0.3947	0.0190
IL-5	−0.3519	0.0382	−0.6168	< 0.0001
IL-10	0.1457	ns	0.1785	ns
MCP-1	−0.1067	ns	−0.2734	ns
YKL-40	−0.4665	0.0047	−0.7473	< 0.0001
IFN-γ	−0.5192	0.0014	−0.6352	< 0.0001
IL-6	−0.476	0.0038	−0.6342	< 0.0001
TGF-β	−0.2106	0.2247	0.0011	ns

The data is presented for markers with adjusted p-values≤0.05 considered significant

*COPD* chronic obstructive pulmonary disorder, *ACO* asthma COPD overlap, ns not significant, *TNF α*s Tumor necrosis factor α, GM-CSF- Granulocyte-macrophage colony-stimulating factor, NGAL-Neutrophil gelatinase-associated lipocalin, MCP 1- Monocyte Chemoattractant Protein-1, YKL 40- Chitinase-3-like protein 1, IFN γ-Interferon γ, TGF β- Transforming growth factor β

### Association between dysregulated metabolites and altered immunological mediators

Pairwise Pearson’s correlation analysis was used to assess the association of the significantly altered metabolites with immunological mediators in serum of ACO patients. Correlation coefficients (r) ranged from 1.0 (maximum positive correlation) to − 1.0 (maximum anticorrelation), with a value of 0 representing no correlation in a heatmap. The red coloured cells represent negative correlations while the blue coloured cells represent positive correlations. The size of the squares indicates the magnitude of the correlation.

**Fig. 4:**
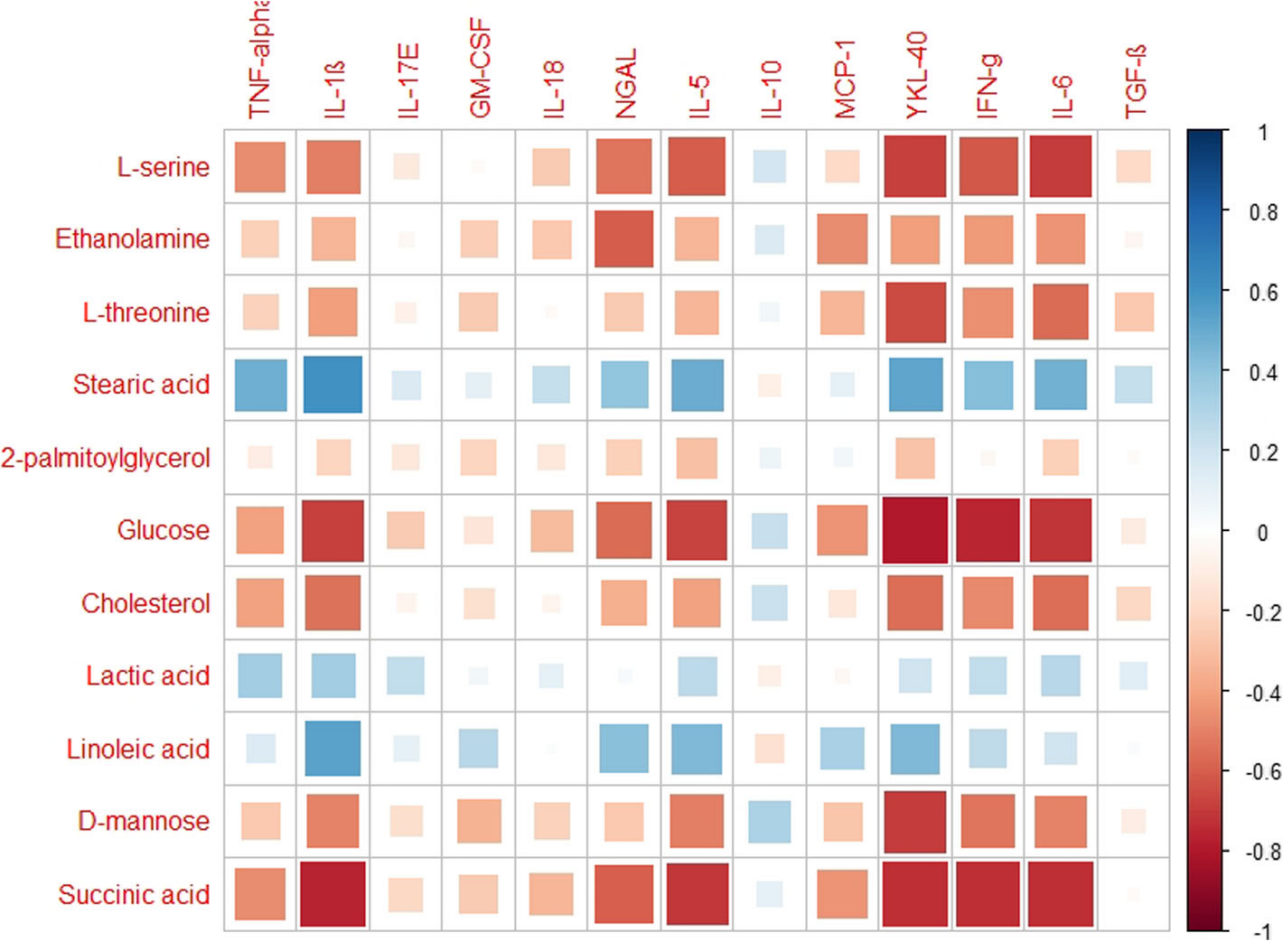
Heatmap is a two-dimensional (2D) correlation matrix which uses monochromatic coloured cells to demonstrate whether two independent variables have a relationship with each other. Heat map demonstrating the Pearson correlation of 13 altered immunological markers with 11 significantly dysregulated metabolites present in serum of ACO patients. Red coloured cells represent negative correlation, and blue coloured cells represent positive correlation. The size of the squares indicates the magnitude of the correlation

Significant negative correlations (− 0.336 to − 0.794, *p* ≤ 0.05) were observed between serine, ethanolamine, threonine, glucose, cholesterol and succinic acid with TNF-α (not significant with ethanolamine, threonine), IL-1β, NGAL (not significant with threonine), MCP-1 (not significant with serine, cholesterol), YKL-40, IFN-γ, and IL-6. Also, a significant negative correlation was observed between succinic acid and IL-18. Mannose too showed negative correlation with IL-1β, GM-CSF, IL-5, YKL-40, IFN-γ and IL-6. In contrast, stearic acid and linoleic acid positively correlated (0.390 to 0.604, p ≤ 0.05) with TNF-α (not significant with linoleic acid), IL-1β, NGAL, IL-5, IFN-γ (not significant with linoleic acid), IL-6 (not significant with linoleic acid) and YKL-40. Lactic acid showed a significant positive correlation with TNF-α and IL-1β (Fig. 4). The energy metabolites and cholesterol negatively correlated with the immunological mediators, whereas the fatty acids and lactic acid showed a positive correlation.

## Discussion

In our earlier study, NMR based metabolomics provided new insights into the altered pathways which could be contributing to the higher mortality and morbidity in ACO in comparison to asthma/COPD [27]. Since MS is known to provide complementary information to NMR [18], serum metabolome of the same patients are analysed using GC-MS. In addition, metabolomic data has been integrated with a wide range of inflammatory mediators to improve the understanding of ACO and effectively differentiate it from asthma, COPD and healthy controls.

A total of 11 metabolites and 13 inflammatory mediators were found to be important in distinguishing ACO from both asthma and COPD. Changes in plasma levels of glucogenic amino acids like serine and threonine have been reported by different groups in both asthma [23] and COPD [38, 39]. Serine and threonine may get converted to pyruvate which in turn enters the TCA cycle to compensate the energy demand [40]. We hypothesize that enhanced cellular demand due to upregulation of glycolysis is responsible for the decrease in the expression levels of these metabolites in ACO.

Sugars such as glucose, mannose and succinate, a TCA cycle intermediate were significantly down-regulated in ACO patients as compared to asthma and COPD. Numerous reports on glucose down-regulation in COPD and asthma exist [22, 41, 42]. A similar trend was also observed in our previous NMR study where significant decrease in glucose level in ACO was observed [27]. Mannose is an intermediate metabolite of galactose metabolism pathway and may be converted to fructose-6-phosphate which enters the glycolytic pathway [43]. The significantly low level of mannose in ACO is attributed to increased glycolytic activity and higher energy demand [44]. Significant decrease in the expression of succinate in ACO cases is in good agreement with the reports of other researchers in COPD and asthma where dysregulated succinate levels have been linked with energy metabolism, hypoxemic stress, or prolonged exertion [45–47].

Ethanolamine, a metabolite of glycerophospholipid metabolism, was also observed to be down-regulated in ACO. Its involvement in the synthesis of phosphatidyl ethanolamine, a central intermediate of lipid metabolism and link with cellular respiration is reported [48]. However, the role of ethanolamine in glucose metabolism per se still remains unclear and warrants further investigation.

The levels of 2-palmitoylglycerol and cholesterol were significantly down-regulated in ACO vs COPD whereas a reverse trend was seen in ACO vs asthma. 2-palmitoylglycerol is a monoacylglycerol and can be associated with lipid cycle disruption with subsequent input to energy cycles [49]. It is also documented that monoacylglycerols are not merely intermediate lipid molecules, but may act as signalling molecules in various inflammatory and other immune system related processes [50]. Cholesterol, on the contrary, has been linked to inflammation, and is reported to decrease in serum of patients with asthma and increase in patients with very severe COPD [51, 52]. The expression pattern of cholesterol in ACO cases may be linked to comprehensive changes in lipid and sterol metabolism.

The increased expression of lactate is attributed to increased glycolysis due to an imbalance in oxygen supplement and demand, as explained by the “Warburg effect”. The concentration levels of lactate have been extensively studied in asthma and COPD and similar mechanisms in ACO are also suggested [24, 52, 53]. Stearic acid (18:0) is a saturated fatty acid (SaFA) which was found to be up-regulated in ACO vs COPD and down-regulated in ACO vs asthma. Numerous reports indicate the involvement of SaFAs with the inflammasome, such as proinflammatory cytokines, VEGF, IL-6, IL-1β etc. [54, 55]. Linoleic acid (9,12- Octadecadienoic acid) also exhibited a similar expression pattern. It plays a critical role in cellular metabolism, signaling and is also the precursor of arachidonic acid, which is actively involved in proinflammatory response and Th2 differentiation in asthma patients [56]. Fatty acid levels are generally related to the metabolic status and diet of the subjects; however, none of the participants in the present study were obese or suffering from any other metabolic disorder. It was also ensured that all subjects followed a similar dietary pattern.

We have also studied a wide range of immunological mediators which have been mostly explored in either asthma and/or COPD. However, no reports exist on the comprehensive immunological profile of ACO. Th1 mediated cytokines such as IFN-γ, IL-12 and IL-2 were estimated in ACO. Only IFN-γ showed significantly altered levels in ACO with respect to asthma, COPD and controls. While IFN-γ is believed to inhibit Th2-mediated inflammation, studies among asthmatic patients have yielded conflicting results, including its association with lung function and disease severity [57–61]. TNFα, at increased levels leads to the development of heightened inflammatory responses in asthma and COPD [14, 62]. It was observed to be higher in ACO subjects in comparison to asthma and controls. COPD cases exhibited the highest circulating levels of TNFα.

The Th2-type cytokines, IL-4, 5, and 13, which are associated with the promotion of IgE and eosinophilic responses mostly in atopy and asthma, and IL-10, characterized by anti-inflammatory response, were explored in ACO [14, 62]. Though not significant, these cytokines were found to be upregulated in ACO, with IL-5 and 10 showing significant changes. Our findings open up the possibility of using a*nti*-*IL*-*5 monoclonal antibodies* for the management of ACO, similar to that of the treatment suggested for severe asthma [63].

IL-25, also known as IL-17E, is evidenced to be involved in airway inflammation in asthma. It promotes and augments allergic Th2 inflammation via production of IL-4, IL-5, and IL-13 [64]. The expression trend of IL 25 in ACO was similar to that of asthma, which may be attributed to its role in systemic inflammation. However, the primary mediators of Th17 cells such as IL-17A and IL-17F were not estimated which restricts us from generating any conclusive ideas regarding Th17 status in ACO cases.

IL-1β has been associated with systemic inflammation in asthma, COPD as well as exacerbations in both the diseases [65, 66]. It is also suggested that raised IgE levels induce IL-1β expression in monocytes which leads to its increased level in blood [67]. ACO patients were found to have significantly higher levels of IL-1β than controls; however, levels were not as high as observed in asthma or COPD. TGF-β is implicated in several aspects of fibrosis, including deposition of extracellular matrix proteins such as collagens and fibronectin [68]. TGF-β levels were highest in COPD patients followed by ACO which is most likely due to the structural changes in the airway epithelium of these patients.

IL-6 plays a key role in acute phase response and is associated with a variety of clinical and biological parameters in asthma, COPD as well as ACO [69, 70]. We found the IL-6 expression level to be the highest in subjects with ACO. This mediator could be useful in clinics for the identification of patients with high systemic inflammation [71]. We also suggest that anti-IL-6 therapies warrant attention as a possible therapeutic strategy for ACO.

IL-18 is known to enhance Th1 response and has a synergistic effect on IL-12 in inducing IFN-γ release and inhibiting Th2 inflammation. We found the highest expression level of IL-18 in COPD patients. This is in good agreement with the findings of Imaoka et al. (2008) [72] where serum levels of IL-18 in COPD patients and smokers were observed to be significantly higher than that of non-smokers. Furthermore, the group also reported a significant negative correlation of IL-18 with FEV_1_ (%) in these patients, which is also in accordance with our observations. IL-18 also has autoimmune regulatory effects on both Th1 and Th2 cytokines [73] and several studies have demonstrated increase in IL-18 activity in Th2 type diseases, such as asthma exacerbations and allergic rhinitis [74].

Other immunological markers such as MCP-1, GM-CSF, YKL-40 and NGAL were also assessed. MCP-1 is evidenced to be higher in blood of both asthma and COPD cases and is strongly related to smoking [75, 76]. GM-CSF is another pleiotrophic and pro-inflammatory cytokine that promotes leucocyte survival and activation, and regulates mucosal immunity and inflammation. YKL-40 a secreted glycoprotein, produced by various cell types, including macrophages, neutrophils, and airway epithelium is reported to be involved in the pathogenesis of COPD, including bronchial neutrophilic airway inflammation and remodelling [77]. It has also been studied in ACO with a few conflicting reports. MCP-1, GM-CSF and YKL-40 were found to be upregulated in ACO with respect to asthma and controls. However, highest expression of these three markers was seen in patients with COPD. NGAL is attributed to activated neutrophils in response to smoke related airway inflammation as well as reactive oxygen species. It is one of the most extensively explored markers of ACO [13, 77, 78]. We found the level to be highest in ACO as compared to asthma, COPD and controls. Our results are in good agreement with reports suggesting higher levels of NGAL in ACO as compared to asthma [13, 78]. Further, a significant negative correlation was observed between serum NGAL and lung function in ACO patients. Our findings are supported by the work of Gao et al. (2016), where an increased NGAL expression in sputum has been independently correlated with degree of airflow limitation in ACO [13]. Other important markers which are frequently studied in asthma and COPD such as periostin, eotaxin and TSLP, though not significant, exhibited an altered profile in ACO.

In recent years, there has been an increasing interest in understanding the group of individuals having features of both asthma and COPD. As the complexity of ACO as a disease entity is gradually unravelled and better understood, a further revision in ACO definition could likely be required [5, 13]. This study in not without limitations. First, owing to the existence of numerous ACO defining guidelines [6–8], the findings of this study are restricted to ACO patients diagnosed as per GINA/GOLD and ATS roundtable diagnostic criteria. Second, this is a single-center study. Genetic variability needs to be accounted for before generalization of the study findings. Similar studies across different countries/continents are, therefore, recommended so that findings on variable ethnicity can be correctly compared. Third, the present findings are limited to patients without active respiratory infections. Alterations in metabolomic profile with lung infections is well realized [79–81]. Since patients with ACO are susceptible to infection [82, 83], it would be worthwhile to investigate ACO metabolomic profiles with and without infections. Fourth, owing to the use of GC-MS platform, this study is limited to the analysis of volatiles with restricted resolution and sensitivity. LC-MS/MS based serum metabolic profiling in the same patient cohort is presently underway. It is envisioned that combining our earlier NMR findings with the complementary GC-MS and LC-MS/MS data will enrich the metabolome coverage and overall improve the data quality. Fifth, in conjunction with serum which reflects analytes in the systemic circulation, it would be useful to analyse the markers at a cellular level in the more proximal biofluid bronchoalveolar lavage fluid (BALF) and lung tissue. However, owing to ethical constraints, this study could not be implemented. Last, our limited sample size may justifiably raise concern regarding robustness of metabolomic data analysis; however, the validation cohort included in this study demonstrates that the dysregulated expression pattern of the metabolites is reproducible and a characteristic of the disease state.

## Conclusion

In conclusion, the present study provides novel insight into metabolic pathways and inflammatory mediators involved in patients with ACO and how these processes are linked to each other and also with the pulmonary function test parameters. Such clinical correlations, on extending to ACO patient phenotypes, could help understand the disease better and aid in tailoring therapies exclusively for ACO.

## Supplementary information

**Additional file 1.**

## Data Availability

The datasets used and/or analyzed during the current study are available from the corresponding author on reasonable request.

## Abbreviations

COPD: Chronic obstructive pulmonary disease
ACO: Asthma COPD Overlap
NMR: Nuclear Magnetic Resonance
MS: Mass Spectrometry
GC/MS: Gas Chromatography/Mass Spectrometry
GINA: Global Initiative for Asthma
GOLD: Global Initiative for Chronic Obstructive Lung Disease
