# Supplementary material for: Metabolomic fingerprinting and systemic inflammatory profiling of asthma COPD overlap (ACO)
Source: Respir Res. 2020 May 24;21:126. doi: 10.1186/s12931-020-01390-4 (PMC7245917; doi:10.1186/s12931-020-01390-4)
Supplement: Supplementary file 1 — Additional file 1. [file 12931_2020_1390_MOESM1_ESM.pdf]

Supplementary Materials

Title: Metabolomic fingerprinting and systemic inflammatory profiling of asthma COPD overlap (ACO)

Authors: Nilanjana Ghosh[a], Priyanka Choudhury[a], Sandeep Rai Kaushik[b], Rakesh Arya[b], Ranjan Nanda[b], Parthasarathi Bhattacharyya[c], Sushmita Roychowdhury[d], Rintu Banerjee[e], Koel Chaudhury[a]*

Affiliations:

[a]School of Medical Science and Technology, Indian Institute of Technology Kharagpur (India)

[b]Translational Health Group, International Centre for Genetic Engineering and Biotechnology New Delhi (India)

[c]Institute of Pulmocare and Research Kolkata (India)

[d]Apollo Gleneagles Hospital Kolkata (India)

[e]Department of Agricultural and Food Engineering, Indian Institute of Technology Kharagpur (India)

Corresponding author: Dr. Koel Chaudhury, Ph.D

Professor, School of Medical Science & Technology (SMST)

Indian Institute of Technology Kharagpur

Kharagpur - 721302, India

Email for all correspondence: koel@smst.iitkgp.ac.in; koeliitkgp@gmail.com

Ph: +913222-283572

Materials and Methods

GC-MS Data Acquisition

Two µl of derivatized serum sample was loaded using splitless mode to RTx-5 column (5% diphenyl, 95% dimethylpolysiloxane; 30 m × 0.25 mm ×0.25 µm; Restek USA) in a GC−MS (7890A GC, 5975 MSD from Agilent Technologies, USA) for separation using an automatic liquid sampler (7683B ALS, Agilent, USA). Helium was used as a carrier gas at a constant flow rate of 1 mL/min. The front inlet temperature was fixed at 250 °C during injection; temperature gradients of 50 to 150 °C (ramp of 10 °C/min) and 150 to 310 °C (ramp of 7 °C/min) with a hold time of 3 min between two ramps and after reaching final temperature were used. Electron ionization (EI) mode was fixed at −70 eV with a scan range of 35 to 600 m/z. Maximum scan speed was 5 spectra/sec with a 6 min solvent delay. The ion-source temperature and quadrupole temperatures were fixed at 230 and 150 °C, respectively. Sample introduction to data acquisition parameters (both GC separation and mass spectrometry) were controlled using ChemStation software (Agilent Technologies, USA), and the run time was 38.43 minutes per sample. Instrument performance over time and metabolite extraction efficiency were evaluated using peak area and retention time of internal standard in samples and quality controls (QCs). Further, as quality check, a mixture of metabolite standards at a known concentration (25 ng/10 µl) was injected after every 8 samples.

Data Pre-processing

Before analysis, the sample codes were opened by a team member not participating in sample processing and GC−MS data acquisition. GC-MS metabolite profiles were processed using Agilent Chemstation data analysis software. Chromatographic processing such as integration and convolution was performed in Agilent ChemStation software using automatic spectral deconvolution (AMDIS) algorithm. The detectable spectral features after background subtraction were annotated using NIST 14 standard mass spectral database (NIST, Gaithersburg, MD). Consistent metabolites with minimum 30% base peak intensity were considered for quantitation. Features with database matching percentage above 80% were only considered for further analysis. After chromatographic integration, peak areas of corresponding metabolites were noted. The peak areas of annotated features were extracted as a data matrix in .CSV file format.

Metabolomic data analysis using multivariate and univariate statistical analysis

Initially, the peak area metabolomic dataset generated post pre-processing was filtered. Features with >50% missing values were removed from the data. The resulting data were

further normalised to constant sum, log transformed and mean scaled using Metaboanalyst 4.0. These data pre-processing strategies such as transformation and scaling help in making features comparable. This normalised data was further subjected to univariate (UVA) and multivariate statistical analysis (MVA).

In order to reveal the pattern and draw conclusion from the metabolomics study, the normalised data files were independently subjected to MVA using SIMCA 13.0.1 software (Umetrics, Sweden) [1]. At first, principal component analysis (PCA) (an unsupervised multivariate statistical approach) was used to generate an overview of data distribution across samples and detect possible outliers. PCA was also performed on the QC samples to ensure the data quality was not compromised. Subsequently, supervised multivariate statistical tools i.e. partial least squares discriminant analysis (PLS-DA) and orthogonal projections to latent structures discriminant analysis (OPLS-DA) were applied to enhance group separation. Parameters including R2 (goodness of the fit), Q2 (predictive ability), and analysis of variance testing of cross validated predictive residuals (CV-ANOVA) score were used to detect robustness of the OPLS-DA model [2,3]. The model robustness was evaluated by performing permutation of the model and comparing it with 200 randomly permutated models. Significant metabolites for group separation in OPLS-DA model were identified using variable importance in the projection (VIP) score. Metabolites with VIP score above 1.3 were considered relevant for group discrimination.

In addition to MVA, UVA was performed to assess statistically significant differences in the expression levels of these metabolites. Statistical significance of the metabolites between the groups was obtained using one way analysis of variance (ANOVA) (Dunnett's post hoc test) or Kruskal–Wallis test (Dunn's post hoc test) (GraphPad Prism version 7.00 for Windows, GraphPad Software, San Diego, CA, USA). Statistical significance was considered to be $p \leq 0.05$. The metabolites were also adjusted for multiple hypothesis testing using FDR correction using Metaboanalyst 4.0. Fold change (FC) was calculated for ACO vs asthma and ACO vs COPD. Metabolites common to both ACO vs COPD and ACO vs asthma which collectively qualify the criteria of VIP, p-value, and FDR were considered significant. These significantly altered metabolites identified in the discovery phase were further validated in a fresh cohort of subjects by performing quantitative UVA post data matrix generation.

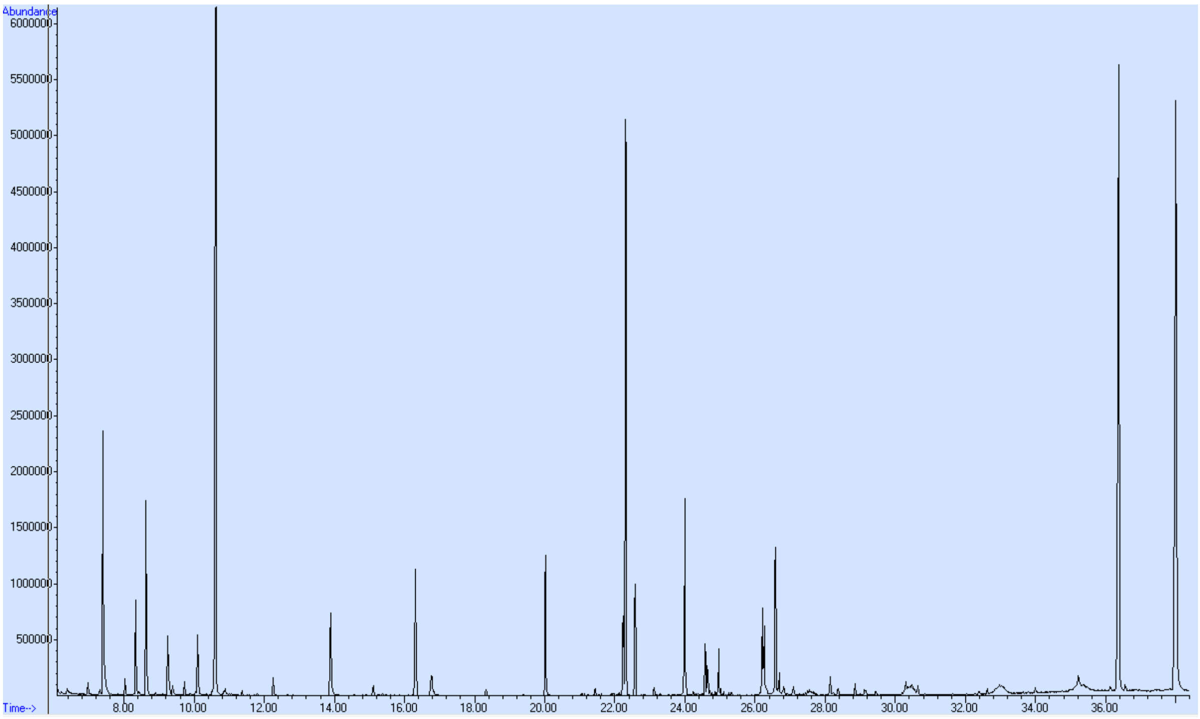

Supplementary Fig. 1. Representative GC/MS chromatogram of serum derived from an ACO patient

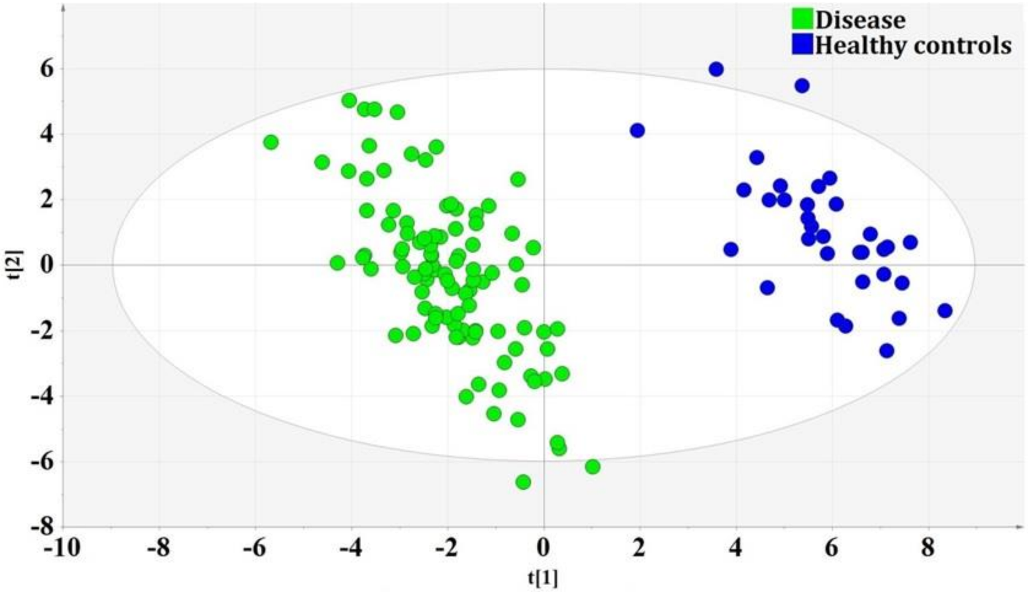

Supplementary Fig. 2. Partial least squares discriminant analysis (PLS-DA) is a supervised multivariate method for assessing relationship between a descriptor matrix X and a response matrix Y. It explains differences between overall class properties. PLS-DA showing optimized discrimination between obstructive lung diseases and healthy controls (R2Y= 0.954 and Q2=0.923, CV-ANOVA=0)

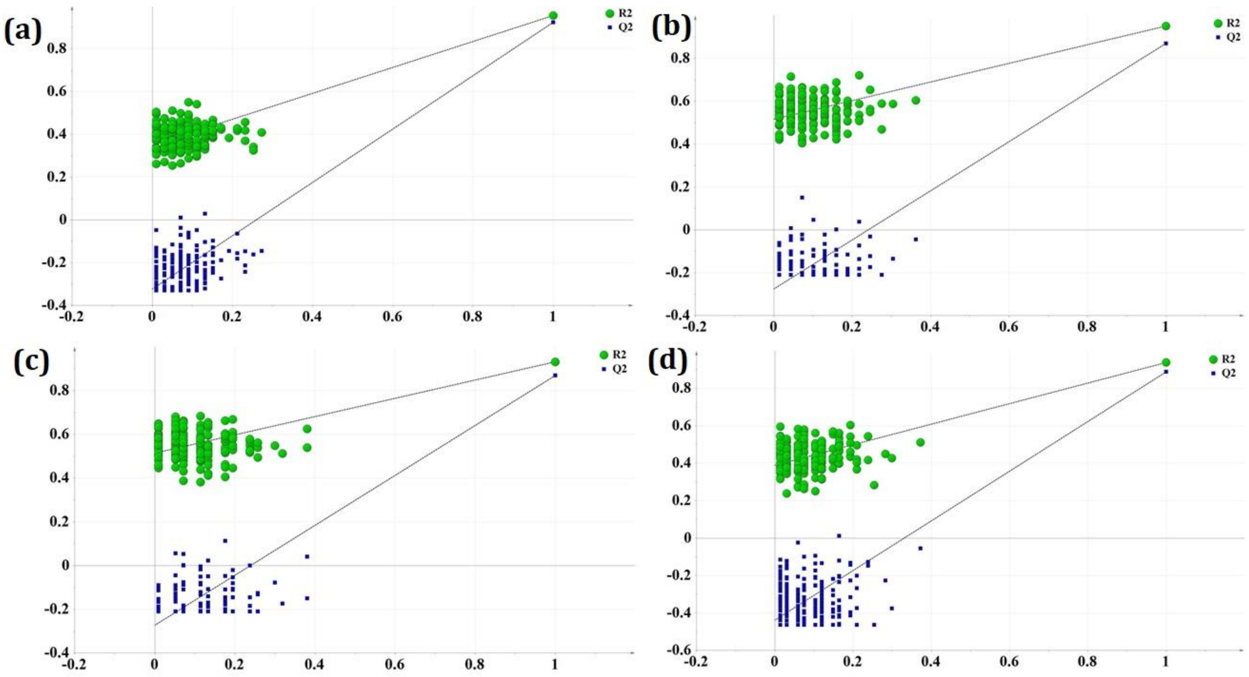

Supplementary Fig. 3. Response permutation test (n=200) to estimate the statistical significance of the partial least squares discriminant analysis (PLS-DA) model. The R2 and Q2 values on the extreme right-hand side of the plot are of the true model, whereas the permutated model parameters are represented on the left-hand side of the plot. The correlation coefficients between true and permutated models represent the X axis. The true class has a correlation of 1.0 with itself. The true model parameters in the validation test exhibited higher values than those of the permutated models which suggests that the generated model can satisfactorily predict ACO better than chance. (a) Healthy controls vs. diseases R2=(0.0,0.349), Q2=(0.0,-0.324) (b) ACO vs. Asthma R2=(0.0,0.515), Q2=(0.0,-0.277) (c) ACO vs. COPD R2=(0.0,0.514), Q2=(0.0,-0.273) (d) ACO, Asthma and COPD R2=(0.0,0.388), Q2=(0.0,-0.439)

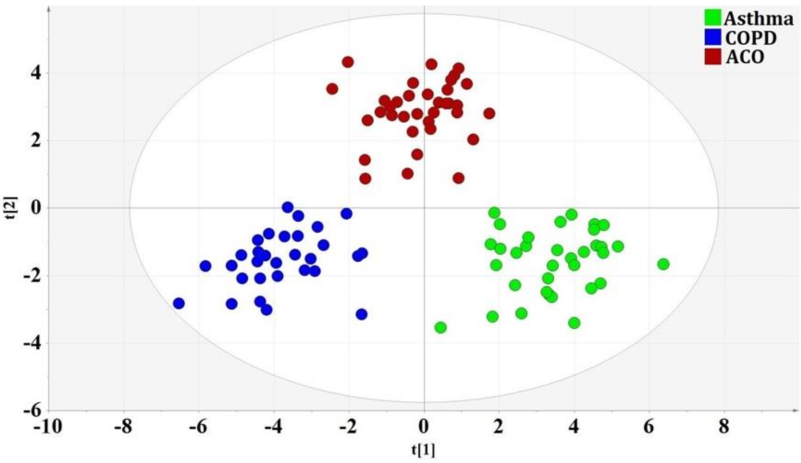

Supplementary Fig. 4. Partial least squares discriminant analysis (PLS-DA) is a supervised multivariate method for assessing relationship between a descriptor matrix X and a response matrix Y. It explains differences between overall class properties. PLS-DA showing optimized discrimination between asthma, COPD and ACO (R2Y= 0.915 and Q2=0.862, CV-ANOVA=0)

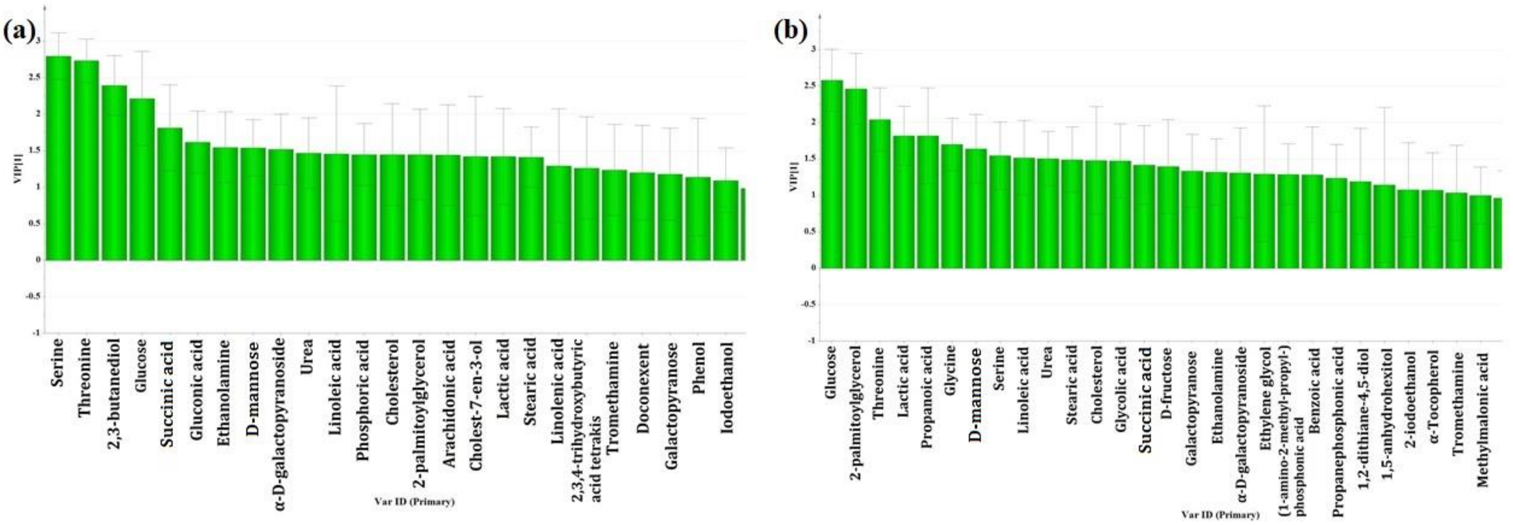

Supplementary Fig. 5. A variable importance in projection (VIP) plot for the orthogonal projections to latent structures discriminant analysis (OPLS-DA) displays the contribution of each metabolite to the models. Features having VIP cut off values >1.30 were identified as the major contributory metabolites responsible for discrimination between (a) ACO and Asthma (b) ACO and COPD

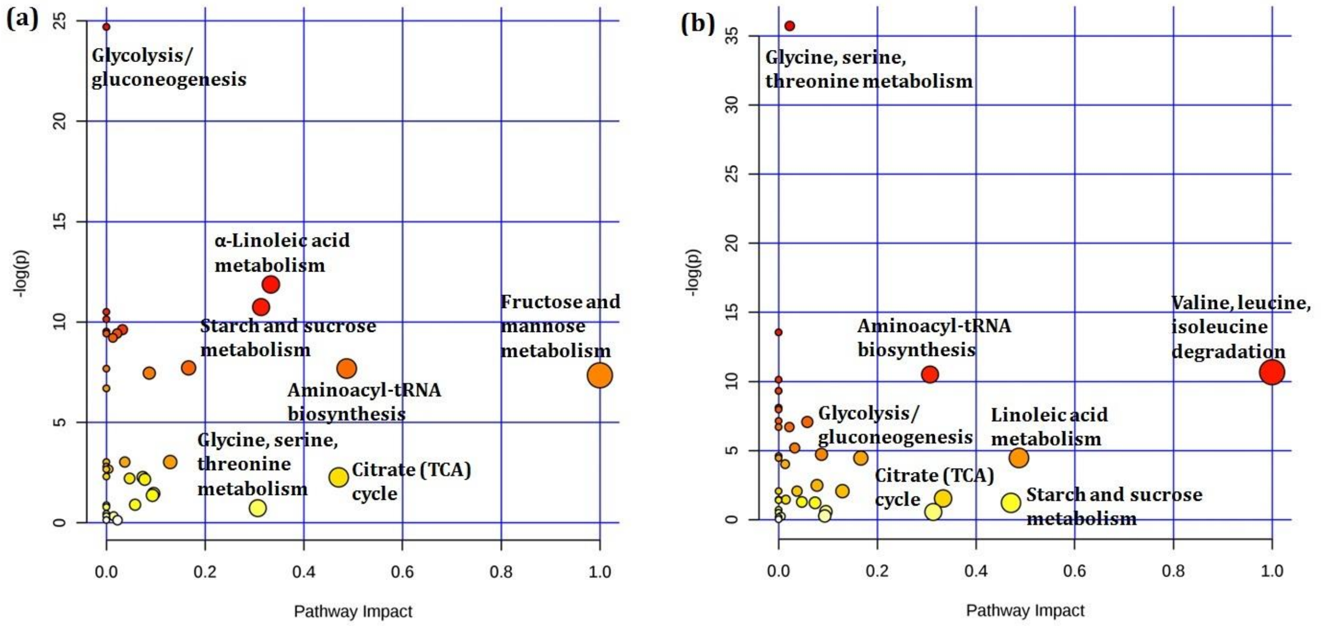

Supplementary Fig. 6. Metabolic pathway analysis (MetPA) of ACO patients. MetPA generated pathway impact score indicates the potential pathways altered in (A) ACO vs. asthma (B) ACO vs. COPD. Degree of importance is represented by node size and colour. Pathway impact represents the potentially altered metabolic pathways. Unaltered pathways have score 0. The color of the circle (yellow to red) is based on the p-value and the radius is defined by the pathway impact values. The highest level of changes in the disease condition is indicated by large red node.

Supplementary Table 1. Pathway analysis using MetPA shows potential pathways involved in ACO vs. Asthma. The table shows the total number of metabolites involved in a pathway, hits, p-values, Holm-adjusted p-values, FDR and impact of the respective pathways.

	Total Cmpd	Hits	Raw p	-log(p)	Holm adjust	FDR	Impact
Fructose and mannose metabolism	20	1	1.88E-11	24.699	7.88E-10	3.94E-10	0
Amino sugar and nucleotide sugar metabolism	37	1	1.88E-11	24.699	7.88E-10	3.94E-10	0
alpha-Linolenic acid metabolism	13	1	7.00E-06	11.87	0.00028	9.80E-05	0.33333
Arachidonic acid metabolism	36	1	2.16E-05	10.745	0.000841	0.000226	0.3135
Neomycin, kanamycin and gentamicin biosynthesis	2	1	2.74E-05	10.506	0.00104	0.00023	0
Alanine, aspartate and glutamate metabolism	28	2	3.95E-05	10.14	0.001461	0.000276	0
Citrate cycle (TCA cycle)	20	1	6.64E-05	9.6202	0.002389	0.000307	0.03273
Arginine biosynthesis	14	1	7.32E-05	9.5227	0.002561	0.000307	0
Purine metabolism	65	1	7.32E-05	9.5227	0.002561	0.000307	0
Cysteine and methionine metabolism	33	1	8.05E-05	9.4273	0.002656	0.000307	0.02184
Sphingolipid metabolism	21	1	8.05E-05	9.4273	0.002656	0.000307	0
Glycerophospholipid metabolism	36	1	9.98E-05	9.212	0.003095	0.000349	0.01324
Aminoacyl-tRNA biosynthesis	48	8	0.000446	7.7152	0.013381	0.001296	0.16667
Glycine, serine and threonine metabolism	33	4	0.000461	7.6824	0.013381	0.001296	0.48704

Valine, leucine and isoleucine biosynthesis	8	4	0.000463	7.6784	0.013381	0.001296	0
Steroid biosynthesis	42	3	0.000578	7.4554	0.015614	0.001518	0.087
Linoleic acid metabolism	5	1	0.000646	7.3455	0.016783	0.001595	1
Glycolysis / Gluconeogenesis	26	1	0.001236	6.6963	0.030888	0.002731	0
Pyruvate metabolism	22	1	0.001236	6.6963	0.030888	0.002731	0
Inositol phosphate metabolism	30	1	0.048484	3.0265	1	0.092561	0.12939
Phosphatidylinositol signaling system	28	1	0.048484	3.0265	1	0.092561	0.03736
Ascorbate and aldarate metabolism	8	1	0.048484	3.0265	1	0.092561	0
Propanoate metabolism	23	3	0.060786	2.8004	1	0.111	0
Steroid hormone biosynthesis	85	1	0.06909	2.6723	1	0.11684	0.00528
Selenocompound metabolism	20	1	0.069548	2.6657	1	0.11684	0
Biosynthesis of unsaturated fatty acids	36	6	0.099591	2.3067	1	0.15615	0
Galactose metabolism	27	4	0.10365	2.2667	1	0.15615	0.07387
Starch and sucrose metabolism	18	2	0.1041	2.2624	1	0.15615	0.47093
Pentose phosphate pathway	22	2	0.10979	2.2092	1	0.15901	0.04712
Arginine and proline metabolism	38	1	0.11514	2.1616	1	0.16119	0.0778
Glutathione metabolism	28	2	0.23359	1.4542	1	0.31648	0.09582
Glycerolipid metabolism	16	1	0.25461	1.368	1	0.33417	0.09346
Primary bile acid biosynthesis	46	2	0.40633	0.90058	1	0.51389	0.05823
Butanoate metabolism	15	2	0.41601	0.87705	1	0.51389	0
Porphyrin and chlorophyll metabolism	30	1	0.4564	0.78438	1	0.54768	0
Glyoxylate and dicarboxylate metabolism	32	4	0.48263	0.72851	1	0.56307	0.30689
Pantothenate and CoA biosynthesis	19	1	0.63989	0.44647	1	0.72636	0
Fatty acid biosynthesis	47	2	0.71935	0.3294	1	0.75568	0.01473
Fatty acid elongation	39	1	0.71969	0.32893	1	0.75568	0
Fatty acid degradation	39	1	0.71969	0.32893	1	0.75568	0
Synthesis and degradation of ketone bodies	5	1	0.86932	0.14004	1	0.87901	0
Valine, leucine and isoleucine degradation	40	4	0.87901	0.12896	1	0.87901	0.02264

Supplementary Table 2. Pathway analysis using MetPA shows potential pathways involved in ACO vs. COPD. The table shows the total number of metabolites involved in a pathway, hits, p-values, Holm-adjusted p-values, FDR and impact of the respective pathways.

	Total Cmpd	Hits	Raw p	-log(p)	Holm adjust	FDR	Impact
Valine, leucine and isoleucine degradation	40	4	3.08E-16	35.715	1.30E-14	1.30E-14	0.02264
Fructose and mannose metabolism	20	1	1.30E-06	13.554	5.33E-05	1.82E-05	0
Amino sugar and nucleotide sugar metabolism	37	1	1.30E-06	13.554	5.33E-05	1.82E-05	0
Linoleic acid metabolism	5	1	2.33E-05	10.669	0.000907	0.000231	1
Glyoxylate and dicarboxylate metabolism	32	4	2.74E-05	10.503	0.001043	0.000231	0.30689
Propanoate metabolism	23	3	3.99E-05	10.13	0.001475	0.000279	0

Arginine biosynthesis	14	1	8.96E-05	9.3199	0.003226	0.000471	0
Purine metabolism	65	1	8.96E-05	9.3199	0.003226	0.000471	0
Glycolysis / Gluconeogenesis	26	1	0.000304	8.0987	0.010333	0.001277	0
Pyruvate metabolism	22	1	0.000304	8.0987	0.010333	0.001277	0
Neomycin, kanamycin and gentamicin biosynthesis	2	1	0.000346	7.9693	0.01107	0.001321	0
Porphyrin and chlorophyll metabolism	30	1	0.000787	7.1477	0.024388	0.002743	0
Primary bile acid biosynthesis	46	2	0.000849	7.0714	0.025472	0.002743	0.05823
Cysteine and methionine metabolism	33	1	0.001228	6.7023	0.035615	0.003439	0.02184
Sphingolipid metabolism	21	1	0.001228	6.7023	0.035615	0.003439	0
Citrate cycle (TCA cycle)	20	1	0.005571	5.1902	0.15041	0.014623	0.03273
Steroid biosynthesis	42	3	0.008848	4.7275	0.23005	0.02186	0.087
Alanine, aspartate and glutamate metabolism	28	2	0.009964	4.6088	0.2491	0.023132	0
Glycine, serine and threonine metabolism	33	4	0.011458	4.4691	0.27499	0.023132	0.48704
Valine, leucine and isoleucine biosynthesis	8	4	0.011553	4.4608	0.27499	0.023132	0
Aminoacyl-tRNA biosynthesis	48	8	0.011566	4.4597	0.27499	0.023132	0.16667
Glycerophospholipid metabolism	36	1	0.017918	4.022	0.37628	0.034207	0.01324
Arginine and proline metabolism	38	1	0.083341	2.4848	1	0.15219	0.0778
Inositol phosphate metabolism	30	1	0.1265	2.0675	1	0.20435	0.12939
Phosphatidylinositol signaling system	28	1	0.1265	2.0675	1	0.20435	0.03736
Ascorbate and aldarate metabolism	8	1	0.1265	2.0675	1	0.20435	0
alpha-Linolenic acid metabolism	13	1	0.21509	1.5367	1	0.32718	0.33333
Fatty acid biosynthesis	47	2	0.23369	1.4537	1	0.32718	0.01473
Fatty acid elongation	39	1	0.2337	1.4537	1	0.32718	0
Fatty acid degradation	39	1	0.2337	1.4537	1	0.32718	0
Biosynthesis of unsaturated fatty acids	36	6	0.24336	1.4132	1	0.32972	0
Pentose phosphate pathway	22	2	0.27644	1.2858	1	0.36282	0.04712
Galactose metabolism	27	4	0.29421	1.2234	1	0.36475	0.07387
Starch and sucrose metabolism	18	2	0.29528	1.2198	1	0.36475	0.47093
Butanoate metabolism	15	2	0.4916	0.7101	1	0.58992	0
Glutathione metabolism	28	2	0.55905	0.58152	1	0.64768	0.09582
Arachidonic acid metabolism	36	1	0.57057	0.56111	1	0.64768	0.3135
Synthesis and degradation of ketone bodies	5	1	0.6028	0.50618	1	0.66625	0
Glycerolipid metabolism	16	1	0.76231	0.2714	1	0.82095	0.09346
Steroid hormone biosynthesis	85	1	0.78623	0.24051	1	0.82554	0.00528
Pantothenate and CoA biosynthesis	19	1	0.83508	0.18022	1	0.85545	0
Selenocompound metabolism	20	1	0.96683	0.033738	1	0.96683	0

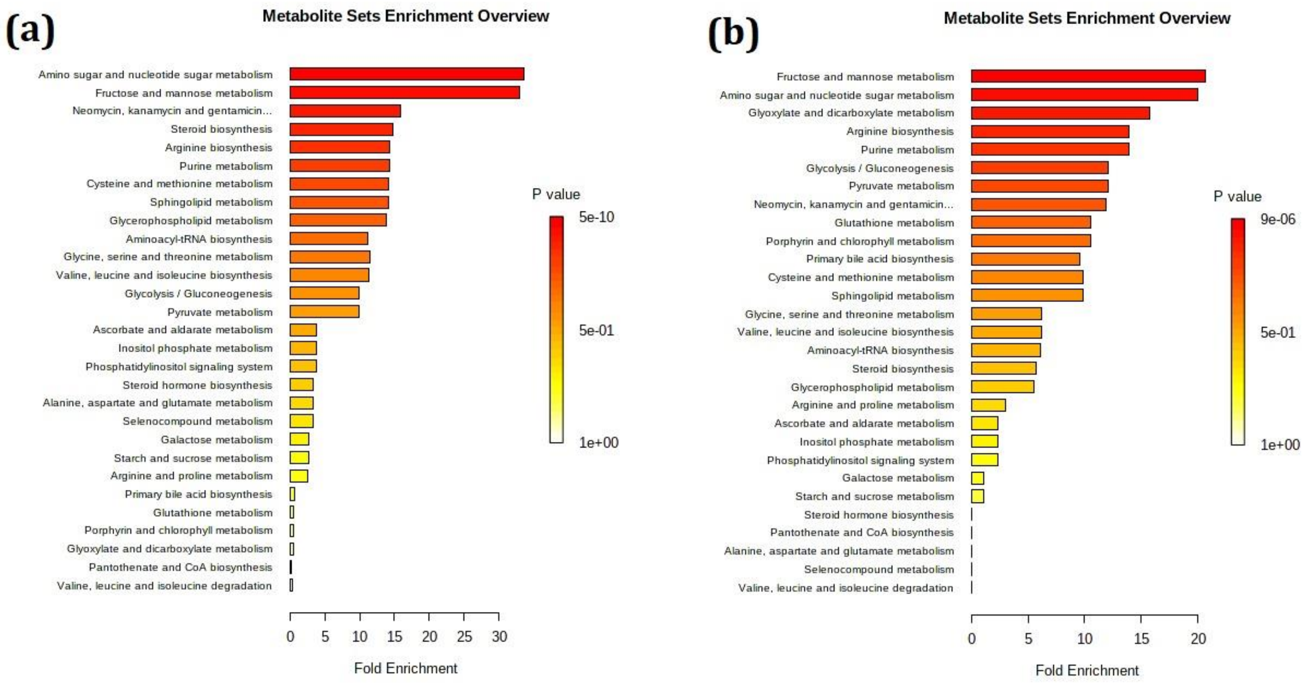

Supplementary Fig. 7. Metabolite set enrichment analysis (MSEA) tool of Metaboanalyst shows the most altered metabolic pathways in the serum of ACO patients as compared to (a) asthma and (b) COPD. Pathways are shown in order of decreasing statistical significance from top to bottom (red to yellow) with length of the bars indicating their estimated fold enrichment

Supplementary Table 3. Data related to the quantitative enrichment analysis (QEA) using metabolite set enrichment analysis (MSEA) tool. Potential pathways involved in ACO vs. asthma shows total compounds, hits, p values and false discovery rates (FDR).

	Total Cmpd	Hits	Statistic Q	Expected Q	Raw p	Holm p	FDR
Amino sugar and nucleotide sugar metabolism	37	1	49.228	1.4706	1.88E-11	5.44E-10	2.75E-10
Fructose and mannose metabolism	20	2	48.349	1.4706	1.90E-11	5.44E-10	2.75E-10
Neomycin, kanamycin and gentamicin biosynthesis	2	1	23.24	1.4706	2.74E-05	0.000739	0.000265
Steroid biosynthesis	42	2	21.664	1.4706	5.50E-05	0.001431	0.000292
Arginine biosynthesis	14	1	21.058	1.4706	7.32E-05	0.001829	0.000292
Purine metabolism	65	1	21.058	1.4706	7.32E-05	0.001829	0.000292
Cysteine and methionine metabolism	33	1	20.843	1.4706	8.05E-05	0.001851	0.000292
Sphingolipid metabolism	21	1	20.843	1.4706	8.05E-05	0.001851	0.000292
Glycerophospholipid metabolism	36	1	20.358	1.4706	9.98E-05	0.002097	0.000322
Aminoacyl-tRNA biosynthesis	48	8	16.457	1.4706	0.000446	0.00892	0.001118
Glycine, serine and threonine metabolism	33	3	16.829	1.4706	0.000461	0.00892	0.001118
Valine, leucine and isoleucine	8	4	16.754	1.4706	0.000463	0.00892	0.001118

biosynthesis							
Glycolysis / Gluconeogenesis	26	1	14.523	1.4706	0.001236	0.021004	0.002559
Pyruvate metabolism	22	1	14.523	1.4706	0.001236	0.021004	0.002559
Ascorbate and aldarate metabolism	8	1	5.6859	1.4706	0.048484	0.72726	0.082708
Inositol phosphate metabolism	30	1	5.6859	1.4706	0.048484	0.72726	0.082708
Phosphatidylinositol signaling system	28	1	5.6859	1.4706	0.048484	0.72726	0.082708
Steroid hormone biosynthesis	85	1	4.8475	1.4706	0.06909	0.82908	0.10085
Alanine, aspartate and glutamate metabolism	28	1	4.8319	1.4706	0.069548	0.82908	0.10085
Selenocompound metabolism	20	1	4.8319	1.4706	0.069548	0.82908	0.10085
Galactose metabolism	27	6	3.909	1.4706	0.1033	0.92971	0.13723
Starch and sucrose metabolism	18	3	3.8939	1.4706	0.1041	0.92971	0.13723
Arginine and proline metabolism	38	1	3.6637	1.4706	0.11514	0.92971	0.14517
Primary bile acid biosynthesis	46	2	1.0648	1.4706	0.40633	1	0.49099
Glutathione metabolism	28	1	0.83064	1.4706	0.4564	1	0.50906
Porphyrin and chlorophyll metabolism	30	1	0.83064	1.4706	0.4564	1	0.50906
Glyoxylate and dicarboxylate metabolism	32	3	0.73866	1.4706	0.48263	1	0.51838
Pantothenate and CoA biosynthesis	19	1	0.32861	1.4706	0.63989	1	0.66274
Valine, leucine and isoleucine degradation	40	3	0.43294	1.4706	0.7444	1	0.7444

Supplementary Table 4. Data related to the quantitative enrichment analysis (QEA) using metabolite set enrichment analysis (MSEA) tool. Potential pathways involved in ACO vs. COPD shows total compounds, hits, p values and false discovery rates (FDR).

	**Total Cmpd**	**Hits**	**Statistic Q**	**Expected Q**	**Raw p**	**Holm p**	**FDR**
Fructose and mannose metabolism	20	2	32.195	1.5625	3.19E-07	9.25E-06	9.25E-06
Amino sugar and nucleotide sugar metabolism	37	1	31.249	1.5625	1.30E-06	3.64E-05	1.88E-05
Glyoxylate and dicarboxylate metabolism	32	3	24.52	1.5625	2.74E-05	0.000741	0.000265
Arginine biosynthesis	14	1	21.765	1.5625	8.96E-05	0.00233	0.00052
Purine metabolism	65	1	21.765	1.5625	8.96E-05	0.00233	0.00052
Glycolysis / Gluconeogenesis	26	1	18.836	1.5625	0.000304	0.007294	0.001254
Pyruvate metabolism	22	1	18.836	1.5625	0.000304	0.007294	0.001254
Neomycin, kanamycin and gentamicin biosynthesis	2	1	18.521	1.5625	0.000346	0.007611	0.001254
Glutathione metabolism	28	1	16.501	1.5625	0.000787	0.016521	0.002239
Porphyrin and chlorophyll metabolism	30	1	16.501	1.5625	0.000787	0.016521	0.002239
Primary bile acid biosynthesis	46	2	15.015	1.5625	0.000849	0.016521	0.002239
Cysteine and methionine metabolism	33	1	15.392	1.5625	0.001228	0.022106	0.00274
Sphingolipid metabolism	21	1	15.392	1.5625	0.001228	0.022106	0.00274
Glycine, serine and threonine metabolism	33	3	9.7149	1.5625	0.011457	0.18331	0.020963

Valine, leucine and isoleucine biosynthesis	8	4	9.6591	1.5625	0.011553	0.18331	0.020963
Aminoacyl-tRNA biosynthesis	48	8	9.4711	1.5625	0.011566	0.18331	0.020963
Steroid biosynthesis	42	2	8.9735	1.5625	0.015269	0.1985	0.026048
Glycerophospholipid metabolism	36	1	8.5756	1.5625	0.017918	0.21501	0.028868
Arginine and proline metabolism	38	1	4.6841	1.5625	0.083341	0.91675	0.1272
Ascorbate and aldarate metabolism	8	1	3.6667	1.5625	0.1265	1	0.16676
Inositol phosphate metabolism	30	1	3.6667	1.5625	0.1265	1	0.16676
Phosphatidylinositol signaling system	28	1	3.6667	1.5625	0.1265	1	0.16676
Galactose metabolism	27	6	1.7589	1.5625	0.29221	1	0.35669
Starch and sucrose metabolism	18	3	1.7379	1.5625	0.29519	1	0.35669
Steroid hormone biosynthesis	85	1	0.11762	1.5625	0.78623	1	0.91203
Pantothenate and CoA biosynthesis	19	1	0.069319	1.5625	0.83508	1	0.93144
Alanine, aspartate and glutamate metabolism	28	1	0.002768	1.5625	0.96683	1	0.97776
Selenocompound metabolism	20	1	0.002768	1.5625	0.96683	1	0.97776
Valine, leucine and isoleucine degradation	40	3	0.058541	1.5625	0.97776	1	0.97776

Supplementary Table 5. Comparisons of serum immunological mediator levels among asthma, COPD ACO and healthy controls. The levels were measured using Magnetic Luminex Assay-Human Premixed Multi-Analyte Kit based on the Luminex xMAP technology. All values are expressed as mean ± standard deviation (SD). One-way ANOVA (Dunnett's post hoc test) or Kruskal–Wallis test (Dunn's post hoc test) was conducted for pairwise comparison

Marker	Mean±SD (pg/ml)	Mean±SD (pg/ml)	Mean±SD (pg/ml)	Mean±SD (pg/ml)	Pairwise p-value		
	ACO	Asthma	COPD	Controls	ACO vs Asthma	ACO vs COPD	ACO vs controls
TNF-α	46.35±8.679	37.66 ±8.864	60.57 ±8.976	35.52 ±8.272	***	****	****
IL-1β	142.3±24.52	158.8 ±25.36	170.1 ±19.15	91.82 ±9.085	**	****	****
IL-17E	2261±420.2	2711 ±433.5	1844 ±270.1	1587 ±208.4	****	****	****
GM-CSF	28.71±5.154	18.55 ±4.485	38.78 ±8.637	14.32 ±4.846	****	****	****
IL-18	1309±399.9	895.7 ±128.9	1808 ±252.3	841.9 ±127.1	****	****	****
NGAL	39.04±3.607	32.19 ±3.796	34.08 ±3.776	30.21 ±3.234	****	****	****
IL-5	56.95±4.926	51.25 ±5.975	47.7 ±5.924	48.46 ±8.382	**	****	****
IL-10	49.26±5.874	37.2 ±5.964	58.8 ±10.42	71.54 ±9.088	****	****	****
MCP-1	1532±133.7	1305 ±199	1951 ±302.4	1085 ±150.3	****	****	****
YKL-40	76883±9091	55073 ±13038	112746 ±20874	47035 ±7641	****	****	****
IFN-γ	95.04±33.47	58.82 ±56.28	151.8 ±56.28	47.26 ±19.3	****	****	****
IL-6	9.239±2.796	5.568 ±1.916	6.155 ±1.487	4.855 ±1.95	****	****	****
TGF-β	754.1±115	664.1 ±124.3	870.5 ±97.9	584.7 ±104.3	**	***	****
IL-12p70	897.2±64.26	806.2 ±67.25	886.7 ±77.53	836.1 ±79.16	*	ns	ns

IL-2	199.8±58.17	326.3 ±49.24	188.3 ±67.08	111 ±51.01	***	ns	*
IL-4	51.35±2.049	53.71 ±5.999	46.85 ±8.022	52.2 ±1.875	ns	*	ns
IL-13	3327±465.8	3593 ±595.8	2255 ±808.7	2107 ±501.7	ns	**	***
IL-1α	102.4±26.12	118.4 ±9.093	158 ±11.67	79.11 ±5.749	ns	****	**
IL-21	206.6±11.85	179 ±18.38	191.6 ±26.38	176.2 ±23.66	*	ns	*
IL-23	1595±428.2	895.5 ±327.3	1307 ±441.8	661.6 ±165.5	**	ns	****
Periostin	599.3±133.2	611.8 ±136.6	381.6 ±114.8	606.8 ±104.9	ns	**	ns
TSLP	23.89±1.726	34.38 ±7.033	20.48 ±3.037	18.83 ±2.787	****	ns	*
IL-8	270.5±81.69	172.3 ±51.45	256.7 ±58.65	106.7 ±17.57	**	ns	****
Eotaxin	1392±383.4	1440 ±241.5	993.2 ±209.2	918.2 ±381.3	ns	*	*

ns-not significant; *$p \leq 0.05$; **$p \leq 0.01$; ***$p \leq 0.0001$

References:

[1] Eriksson L, Byrne T, Johansson E, Trygg J, Vikström C. Multi-and megavariate data analysis basic principles and applications. Umetrics Academy; 2013.

[2] Westerhuis JA, Hoefsloot HC, Smit S, Vis DJ, Smilde AK, van Velzen EJ, van Duijnhoven JP, van Dorsten FA. Assessment of PLSDA cross validation. Metabolomics. 2008; 4(1): 81-9. https://doi.org/10.1007/s11306-007-0099-6.

[3] Trygg J, Wold S. Orthogonal projections to latent structures (O-PLS). J Chemom 2002; 16(3): 119-28. https://doi.org/10.1002/cem.695.
